# Roasting-induced changes in the metabolite profile of laver (*Neopyropia yezoensis*): Focus on mycosporine-like amino acids and antioxidant activity

**DOI:** 10.1016/j.fochx.2026.104450

**Published:** 2026-09-19

**Authors:** Si-Hun Song, Seung-Hun Chae, Seung-Sik Cho, Hyung Gyun Kim, Jeong-Yong Cho

**Affiliations:** a Department of Integrative Food, Bioscience and Biotechnology, Chonnam National University, Gwangju 61186, Republic of Korea; b Department of Horticulture and Interdisciplinary Program in IT-Bio Convergence System, Chonnam National University, Gwangju 61186, Republic of Korea; c Department of Pharmacy and Biomedicine, Health & Life Convergence Sciences, BK21 FOUR, College of Pharmacy, Mokpo National University, Muan 58554, Jeonnam, Republic of Korea; d Institute of Natural BIO Industry for Namwon, 43 Simyo-gil, Namwon-si, Jeonbuk-do, 55801, Republic of Korea

**Keywords:** UPLC-QTOF-MS, Metabolomics, Thermal processing, Thermal dehydration

## Abstract

This study investigated non-volatile metabolites in laver (*Neopyropia yezoensis*) roasted under different temperature/time conditions. Ultra-performance liquid chromatography–quadrupole time-of-flight mass spectrometry-based metabolomics identified 69 metabolites, including mycosporine-like amino acids (MAAs) and their dehydrated derivatives. Multivariate analysis showed that dehydrated MAAs were among the key roasting-responsive metabolites. As roasting intensity increased, MAAs decreased to 0.08-fold of the initial value, while dehydrated MAAs increased 1.22- to 13.61-fold. Antioxidant activity increased, with ABTS^+^ radical scavenging activity and FRAP exhibiting maximum increases of 4.55- and 4.18-fold, respectively. Pearson's correlation analysis revealed strong positive associations (*r* > 0.93) between dehydrated MAAs and antioxidant activity. This pattern is consistent with the extended conjugation and enhanced electron delocalization of dehydrated MAAs, which may facilitate radical stabilization. These findings provide insight into metabolite and antioxidant activity changes during roasting and offer fundamental data for optimizing roasting conditions.

## 1 Introduction

Laver (*Neopyropia* and *Neoporphyra* spp.), a representative red alga, has been increasingly recognized for its functional properties. Consequently, the consumption and production of laver have shown a continuous upward trend. In recent years, interest in laver as a low-calorie health food has grown not only in Asia but also in the West (Kim et al., 2024). Laver contains a variety of bioactive compounds, including polysaccharides, proteins, minerals, vitamins, mycosporine-like amino acids (MAAs), and polyunsaturated fatty acids (PUFAs) (Kim et al., 2021). These compounds have been reported to exert diverse physiological activities, including cholesterol-lowering, anti-inflammatory, antioxidant, anticancer, and antihypertensive effects (Cho & Rhee, 2019).

MAAs, a major class of functional compounds in laver, are secondary metabolites composed of a central cyclohexenone or cyclohexenimine ring structure conjugated to amino acids, amino alcohols, or their derivatives. Among them, shinorine and porphyra-334 are known as the main compounds in laver (Choi et al., 2022). MAAs are primarily recognized as naturally occurring UV-absorbing compounds (Peng et al., 2023); however, their structures and antioxidant activities may also change during thermal processing. In particular, heat treatment at temperatures above 100 °C has been reported to convert porphyra-334 in *Neopyropia yezoensis* (formerly *Porphyra yezoensis*) into dehydrated porphyra-334, which exhibits enhanced diphenylpicrylhydrazyl (DPPH) radical-scavenging activity (Yoshiki et al., 2009). Similarly, heat-induced dehydration of palythine and porphyra-334 from *Palmaria palmata* increased their ABTS^+^ radical scavenging activity (Nishida et al., 2022).

Laver has a high moisture content when fresh, which makes it highly susceptible to microbial spoilage and decay. It is therefore generally dried to maintain its quality attributes during long-term storage. Laver dried by heat treatment has been utilized in various processed foods, such as seasoned laver, roasted laver, and snacks (Choi et al., 2022). Heat treatment influences the flavor and composition of foods by inducing chemical changes, such as protein denaturation, the Maillard reaction, and lipid oxidation, which become particularly pronounced at temperatures above 90 °C (Rabbani et al., 2025). More broadly, thermal-processing conditions—including temperature and treatment intensity—strongly influence product characteristics and quality outcomes in heat-treated foods (EL-Mesery, Kamel and Emara, 2021). Generally, lipid oxidation and the degradation of heat-sensitive bioactive compounds proceed more rapidly as roasting temperature and time increase, potentially altering the overall compound profile. Therefore, identifying compound changes induced by heat treatment is essential for improving the quality and functionality of processed laver products.

To date, most studies have focused on the proximate compositions, metabolites, quality factors, and physicochemical properties of raw or dried laver (Kim et al., 2021; Kim et al., 2025; Lee et al., 2025; Yan et al., 2025), whereas investigations on changes in non-volatile metabolites, the behavior of MAAs, and antioxidant activity during roasting remain limited. In particular, although consumption of heat-processed laver products, such as roasted laver, has been steadily increasing in recent years, fundamental research on their chemical constituents remains insufficient (Lee & Lee, 2024). The drying process involving heat treatment has been reported as an important factor influencing not only the metabolite profile of laver but also its sensory attributes, including flavor (Yan et al., 2025).

Yoshiki et al. (2009) demonstrated the heat-induced dehydration of porphyra-334 and reported enhanced DPPH radical-scavenging activity of its dehydrated derivative. However, their study primarily focused on this specific MAA transformation. In contrast, this study aimed to systematically characterize changes in non-volatile metabolites across multiple roasting temperature–time conditions using ultra-performance liquid chromatography–quadrupole time-of-flight mass spectrometry (UPLC-QTOF-MS). Specifically, we tracked individual MAAs and their dehydrated derivatives, including additional dehydrated MAA candidates that were tentatively assigned based on MS/MS data, and examined the associations between roasting-induced metabolite changes and in vitro antioxidant activity. Collectively, this integrated approach provides a broader understanding of heat-induced compositional changes in roasted laver and contributes to the optimization of its processing conditions.

## 2 Materials and methods

### 2.1 Preparation of roasted laver samples

*N. yezoensis* was collected from Wando County (34°19′09.2″ N, 126°44′46.3″ E), Korea, in January 2023. Laver was washed, shredded, and molded into sheets (20 × 20 cm), then dried at 70 °C to a final moisture content of 9.0 ± 1.0%. These dried sheets, without further roasting, served as the control. Roasted laver samples were then prepared under various roasting conditions using a commercial laver roaster (NY-2698, Kawashima, Nagoya, Japan). The roasting temperatures were set at 160, 180, and 200 °C, and roasting times were 1, 5, 10, 15, and 20 s, respectively (Table S1). For each roasting condition, separate laver sheets originating from the same collection batch were roasted and processed independently. Thus, the experimental replicates captured within-batch variation resulting from roasting and sample preparation but did not account for biological variation among independent collection batches. After roasting, samples were ground using a grinder (SFM-555SP, Shinil, Cheonan, Korea), thoroughly mixed to ensure homogeneity, and stored at −80 °C until further analysis.

### 2.2 Preparation of roasted laver extract

Roasted laver powder (100 mg) was extracted with 20 mL of a water/methanol mixture (50:50, *v*/v) at 25 °C for 24 h. The extracts were centrifuged at 4000 rpm for 10 min at 4 °C, and the supernatants were collected and filtered through a 0.2 μm syringe filter (Whatman, Kent, England). The filtrates were used for metabolite profiling and antioxidant activity assays. For each roasting condition, three independent extractions were performed using the homogenized sample powder.

### 2.3 Liquid chromatography-mass spectrometry (LC-MS)-based metabolite analysis

Metabolite profiling of roasted laver was performed using an ACQUITY UPLC™ system (Waters, Milford, MA, USA) with an ACQUITY UPLC HSS T3 column (1.8 μm, 2.1 × 50 mm; Waters) and coupled with a Xevo G2-XS quadrupole time-of-flight mass spectrometer (Waters). The mobile phases consisted of distilled water (A) and acetonitrile (B), both containing 0.1% formic acid. The gradient elution condition was 0–2 min, 0% B; 2–5 min, 30% B; 5–7 min, 60% B; 7–11 min, 70% B; and 11–13 min, 100% B. The column temperature was maintained at 40 °C, and the flow rate was set to 0.35 mL/min. Mass spectrometry (MS) analysis was carried out in both positive and negative electrospray ionization (ESI) modes. The parameters were as follows: capillary voltage, 2.5 kV; scan range, *m/z* 40–1200; cone voltage, 40 V; cone gas flow, 50 L/h; desolvation gas flow, 600 L/h; desolvation temperature, 200 °C; and source temperature, 130 °C. Leucine–enkephalin (positive mode, *m/z* 556.2771; negative mode, *m/z* 554.2615) was used as the lock mass. Each roasted laver sample was analyzed in triplicate. Stability of the analytical instrument was evaluated using a pooled quality control (QC) sample prepared by mixing equal aliquots of all extracts. Accurate mass measurements and elemental composition of detected compounds were obtained using MassLynx 4.2 software (Waters). Metabolite identification was performed by comparing the obtained mass spectra data with those in online databases, including MassBank (https://massbank.eu/MassBank/) and LIPID MAPS (https://www.lipidmaps.org/), as well as published references. Metabolite annotation followed the Metabolomics Standards Initiative (MSI) guidelines. Identification confidence was classified as follows: Level 1 (identified compounds) was assigned when retention time and mass spectra matched those of authentic standards; Level 2 (putatively annotated compounds) was assigned based on spectral matches with public databases or literature; and Level 3 (putatively characterized compound classes) was assigned based on spectral similarity to known chemical classes.

### 2.4 Antioxidant activity assay

#### 2.4.1 ABTS^+^ radical scavenging activity

The antioxidant activity of roasted laver was evaluated using a modified method described by Thaipong et al. (2006). For the preparation of the ABTS^+^ radical solution, 7 mM ABTS ammonium was dissolved in potassium phosphate buffer (pH 7.4) and reacted with 2.45 mM potassium persulfate. The flask containing the mixture was wrapped in aluminum foil and kept at room temperature (25 °C) for 12–16 h until a deep blue color developed. The solution was then diluted with potassium phosphate buffer until the absorbance at 734 nm reached 1.0 ± 0.02. Subsequently, 190 μL of the prepared ABTS^+^ solution was mixed with 10 μL of roasted laver extract and incubated in the dark at room temperature for 30 min. The absorbance was measured at 734 nm using a microplate reader (MQX200R, BioTek, Winooski, VT, USA). A calibration curve was constructed with ascorbic acid (Y = −0.3505× + 0.9718, R^2^ = 0.997) at concentrations of 0.1–2.5 mg/mL. ABTS^+^ radical scavenging activity of the samples was presented as milligrams of ascorbic acid equivalents per 100 g dry weight (mg AAE/100 g dw).

#### 2.4.2 Ferric reducing antioxidant power

The ferric reducing antioxidant power (FRAP) of roasted laver extract was determined based on the reduction of the ferric (Fe^3+^)–2,4,6-tripyridyl-*S*-triazine (TPTZ) complex to the ferrous (Fe^2+^) form by antioxidants. The FRAP working solution was freshly prepared by mixing 2.5 mL of 10 mM TPTZ solution in 40 mM HCl with 2.5 mL of 20 mM FeCl₃·6H₂O, followed by dilution in 300 mM acetate buffer (pH 3.6). The mixture was incubated at 37 °C for 30 min, and absorbance was measured at 593 nm. For the assay, 190 μL of the FRAP solution was mixed with 10 μL of roasted laver extract and incubated in the dark at room temperature (25 °C) for 30 min. Absorbance was then recorded at 593 nm using a microplate reader. A calibration curve was constructed using ascorbic acid (Y = 0.5362× + 0.0699, R^2^ = 0.9956) at concentrations of 0.1–2.5 mg/mL, and FRAP values were presented as described in Section 2.4.1.

### 2.5 Statistical analysis

MAA levels and antioxidant activities were analyzed using SPSS software (version 29; IBM, Chicago, IL, USA). Two-way analysis of variance (ANOVA) was used to evaluate the main effects of roasting temperature and roasting time, as well as their interaction. Tukey's multiple comparison test was applied for post hoc comparisons. Statistical significance was set at *p* < 0.05. Additionally, Dunnett's test was performed to compare each roasted treatment with the unroasted control. All measurements were performed in at least triplicate. Results are presented as means ± standard deviations (SD).

LC-MS raw data were processed with Progenesis QI software (Waters) peak alignment and peak picking to generate a metabolite matrix. Multivariate statistical analyses were performed using MetaboAnalyst 6.0 (https://www.metaboanalyst.ca/). Features with a relative standard deviation (RSD) > 20% in pooled QC samples were excluded. Data normalization was performed using square root transformation, followed by Pareto scaling.

Metabolite trends in roasted laver according to roasting temperature and time were initially explored using principal component analysis (PCA) and loading plots. ANOVA-simultaneous component analysis (ASCA) was then applied to determine the relative contributions of roasting temperature, roasting time, and their interaction to the metabolome profile of roasted laver. Permutation testing with 200 permutations was used to reduce the possibility of chance effects. In the ASCA model, leverage was used to assess the importance of each metabolite to the factor submodel, while the squared prediction error (SPE) was used to evaluate the goodness of fit for each metabolite. Variables with high leverage and low SPE were considered influential contributors to the corresponding factor model.

For feature-wise statistical validation, two-way ANOVA was performed on the identified metabolites to test the effects of roasting temperature, roasting time, and their interaction. The resulting *p*-values were adjusted using the Benjamini–Hochberg false discovery rate (FDR) procedure, and metabolites with *q* < 0.05 were considered significant. Heatmap visualization and hierarchical clustering analysis (HCA) were used to examine variation patterns in significant metabolites across roasting temperature and time conditions. HCA and heatmap analysis were performed based on Spearman's distance measure and Ward's linkage algorithm. Pearson's correlation analysis was conducted using GraphPad Prism (version 5.0; GraphPad Software, San Diego, CA, USA).

## 3 Results and discussion

### 3.1 Identification of key metabolites and dehydrated MAAs in roasted laver

Untargeted metabolomic profiling of roasted laver prepared under different roasting temperatures and times was performed using UPLC-QTOF-MS. Analyses were carried out in both positive and negative ion modes to obtain comprehensive and reliable metabolite information. Representative LC-MS chromatograms acquired in both positive and negative ion modes are shown in Fig. S1. A total of 69 major metabolites were identified in roasted laver, including 11 amino acids and their derivatives, 3 organic acids, 7 MAAs, 11 nucleic acid-related compounds, 24 phospholipids, 2 glycolipids, 5 other compounds, and 6 dehydrated MAAs (Table 1). These compounds were identified based on their precursor ions and MS/MS fragmentation patterns, by comparison with spectra in databases such as MassBank and LIPID MAPS, and by reference to our previous study (Song et al., 2025). In particular, comparison of the LC-MS (positive ion mode) total ion chromatograms of dried unroasted (control) and roasted laver extracts (Fig. S2) revealed that the peak intensities of major MAAs, including shinorine and porphyra-334, were higher in the control. Moreover, the peak intensities of these MAAs gradually decreased with increasing roasting temperature and time, while six additional peaks detected at retention times 3.92–5.10 min increased in intensity.

**Table 1:** MS results of metabolites identified in roasted laver.

No.	Metabolite	RT	Adduct	Precursor ion (*m/z*)		Mass error (ppm)	Chemical formula	Fragment ion (*m/z*)	MSI levels
		(min)		Calculated mass	Observed mass				
Amino acids and derivatives									
1	Taurine	0.68	[M + H]^+^	126.0225	126.0221	−3.2	C_2_H_7_NO_3_S	108.0120	1
2	Aspartic acid	0.68	[M + H]^+^	134.0453	134.0450	−2.2	C_4_H_7_NO_4_	116.0353 / 88.0399 / 74.0233	1
3	Alanine	0.70	[M + H]^+^	90.0555	90.05510	−4.4	C_3_H_7_NO	44.0498	1
4	Choline	0.70	[M]^+^	104.1075	104.1073	−1.9	C_5_H_14_NO	60.0808 / 58.0649	1
5	Glutamic acid	0.72	[M + H]^+^	148.0610	148.0605	−3.4	C_5_H_9_NO_4_	130.0499 / 102.0550 / 84.0446	1
9	Citrulline	0.75	[M + H]^+^	176.1035	176.1030	−2.8	C_6_H_13_N_3_O_3_	159.0770 / 113.0704	1
11	Acetylcholine	0.84	[M]^+^	146.1181	146.1179	−1.4	C_7_H_16_NO_2_	87.0442 / 60.0810 / 58.0650	1
16	2,6-Piperidinedicarboxylicacid	1.16	[M + H]^+^	174.0766	174.0765	−0.6	C_7_H_11_NO_4_	128.0707 / 82.0652 / 55.0544	2
25	Pyroglutamic acid	1.90	[M + H]^+^	130.0504	130.0503	−0.8	C_5_H_7_NO_3_	84.0447	1
27	Tyrosine	2.55	[M + H]^+^	182.0817	182.0814	−1.6	C_9_H_11_NO_3_	136.0758	1
40	Tryptophan	4.53	[M + H]^+^	205.0977	205.0975	−1.0	C_11_H_12_N_2_O_2_	188.0710 / 146.0607 / 118.0653	1
	
Mycosporine-like amino acids (MAAs)									
12	Shinorine	0.85	[M + H]^+^	333.1298	333.1295	−0.9	C_13_H_20_N_2_O_8_	318.1051 / 303.1186 / 255.0978 / 230.1257 / 197.0920 / 186.1000	1
13	Palythine	0.85	[M + H]^+^	245.1137	245.1133	−1.6	C_10_H_16_N_2_O_5_	230.0913 / 227.1025 / 209.0920 / 186.1001	2
18	Asterina-330	1.25	[M + H]^+^	289.1400	289.1407	2.4	C_12_H_20_N_2_O_6_	274.1142 / 243.0959 / 230.1263 / 212.1215 / 197.0921 / 186.0998	2
24	Porphyra-334	1.86	[M + H]^+^	347.1454	347.1451	−0.9	C_14_H_22_N_2_O_8_	332.1220 / 303.1190 / 288.1312 / 227.1027 / 200.1154 / 186.0998	1
28	Mycosporine-glycine-alanine	2.72	[M + H]^+^	317.1349	317.1345	−1.3	C_13_H_20_N_2_O_7_	302.1123 / 258.1189 / 214.1313 / 186.1004	2
30	Aplysiapalythine B	3.11	[M + H]^+^	273.1450	273.1450	0.0	C_12_H_20_N_2_O_5_	258.1216 / 214.1314	2
35	Usujirene	4.08	[M + H]^+^	285.1450	285.1456	2.1	C_13_H_20_N_2_O_5_	270.1205 / 241.0820 / 226.1305 / 197.0924	2
	
Organic acids									
14	Malic acid	1.03	[M-H]^−^	133.0137	133.0139	1.5	C_4_H_6_O_5_	115.0039 / 71.0140	1
21	Citric acid	1.74	[M-H]^−^	191.0192	191.0194	1.0	C_6_H_8_O_7_	111.0088 / 87.0081 / 85.0290	1
26	Succinic acid	2.27	[M-H]^−^	117.0188	117.0191	2.6	C_4_H_6_O_4_	99.0077 / 73.0289	1
	
Nucleic acid-related compounds									
15	5’-CMP	1.07	[M-H]^−^	323.0280	323.0286	1.9	C_9_H_14_N_3_O_8_P	138.9775 / 96.9687 / 78.9592	1
17	5’-UMP	1.22	[M-H]^−^	323.0280	323.0284	1.2	C_9_H_13_N_2_O_9_P	211.0006 / 111.0206 / 96.9686 / 78.9589	1
19	5’-AMP	1.43	[M + H]^+^	348.0709	348.0701	−2.3	C_10_H_14_N_5_O_7_P	136.0622 / 97.0283	1
20	Cytidine	1.48	[M-H]^−^	242.0777	242.0770	−2.9	C_9_H_13_N_3_O_5_	109.0398	1
22	5’-GMP	1.75	[M + H]^+^	364.0658	364.0657	−0.8	C_10_H_14_N_5_O_8_P	152.0587	1
23	5’-IMP	1.77	[M-H]^−^	347.0393	347.0407	4.0	C_10_H_13_N_4_O_8_P	211.0013 / 135.0314 / 96.9681 / 92.0258 / 78.9589	1
29	Uridine	2.73	[M + Na]^+^	267.0593	267.0591	−0.7	C_9_H_12_N_2_O_6_	113.0325	1
31	Adenosine	3.82	[M + H]^+^	268.1046	268.1042	−1.5	C_10_H_13_N_5_O_4_	136.0622 / 119.0355	1
32	Guanosine	3.83	[M-H]^−^	282.0838	282.0835	−1.1	C_10_H_13_N_5_O_5_	150.0410 / 133.0148 / 108.0198	1
33	Inosine	3.84	[M-H]^−^	267.0729	267.0728	−0.4	C_10_H_12_N_4_O_5_	135.0308 / 108.0212	1
38	5′-Methylthioadenosine	4.46	[M + H]^+^	298.0974	298.0977	1.0	C_11_H_15_N_5_O_3_S	136.0624 / 119.0355	2
	
Dehydrated MAAs									
34	Dehydrated palythine	3.92	[M + H]^+^	227.1032	227.1033	0.4	C_10_H_14_N_2_O_4_	212.0791 / 209.0927 / 197.0927 / 194.0691 / 181.0977 / 168.0894 / 166.0744 / 163.0871 / 151.0870	3
36	Dehydrated asterina-330	4.33	[M + H]^+^	271.1294	271.1294	−3.0	C_12_H_18_N_2_O_5_	256.1049 / 253.1180 / 241.1181 / 238.0942 / 225.1227 / 221.0918 / 212.1149 / 210.1009 / 195.1124	3
37	Dehydrated aplysiapalythine B	4.43	[M + H]^+^	255.1345	255.1343	−0.8	C_12_H_18_N_2_O_4_	240.1109 / 237.1231 / 225.1238 / 222.1004 / 209.1289 / 196.1210 / 194.1053 / 191.1181 / 179.1182	3
39	Dehydrated shinorine	4.47	[M + H]^+^	315.1192	315.1193	0.3	C_13_H_18_N_2_O_7_	315.1191 / 297.1079 / 285.1085 / 269.1130 / 265.0816 / 251.1031 / 239.1029 / 237.0872	3
41	Dehydrated porphyra-334	4.84	[M + H]^+^	329.1349	329.1348	−0.3	C_14_H_20_N_2_O_7_	311.1234 / 299.1235 / 283.1284 / 279.0975 / 265.1172 / 253.1162 / 251.1005 / 235.1065 / 233.0927	2
42	Dehydrated mycosporine-glycine-alanine	5.10	[M + H]^+^	299.1243	299.1239	−1.3	C_13_H_18_N_2_O_6_	284.1012 / 281.1131 / 269.1133 / 266.0880 / 253.1183 / 251.1008 / 235.1086 / 223.1058 / 221.0905	3
	
Phospholipids									
44	LPC 18:4	8.27	[M + H]^+^	516.3090	516.3079	−2.1	C_26_H_46_NO_7_P	184.0735 / 104.1076 / 86.0962	2
45	LPC 14:0	8.55	[M + H]^+^	468.3090	468.3083	−1.5	C_22_H_46_NO_7_P	184.0736 / 104.1073 / 86.0968	2
46	LPE 20:5	8.63	[M-H]^−^	498.2621	498.2617	−0.8	C_25_H_42_NO_7_P	301.2165 / 257.2260 / 214.0475 / 196.0379 / 140.0113	2
47	LPC 20:5	8.71	[M + H]^+^	542.3247	542.3245	−0.4	C_28_H_48_NO_7_P	524.3130 / 184.0737 / 104.1074 / 86.0966	2
48	LPC 18:3	8.79	[M + H]^+^	518.3247	518.3233	−2.7	C_26_H_48_NO_7_P	184.0735 / 104.1073 / 86.0967	2
49	LPE 18:2	9.22	[M-H]^−^	476.2777	476.2759	−3.8	C_23_H_44_NO_7_P	279.2321 / 196.0380 / 140.0118	2
50	LPE 20:4	9.27	[M-H]^−^	500.2777	500.2771	−1.2	C_25_H_44_NO_7_P	303.2319 / 259.2412 / 214.0472 / 196.0357 / 140.0115	2
51	LPI 20:5	9.29	[M-H]^−^	617.2727	617.2719	−1.3	C_29_H_47_O_12_P	315.0473 / 301.2174 / 171.0026 / 152.9956	2
52	LPC 18:2	9.37	[M + H]^+^	520.3403	520.3397	−1.2	C_26_H_50_NO_7_P	184.0733 / 104.1072 / 86.0959	2
53	LPE 16:1	9.37	[M-H]^−^	450.2621	450.2606	−3.3	C_21_H_42_NO_7_P	253.2127 / 214.0478 / 196.0416 / 140.0114	2
54	LPC 20:4	9.39	[M + H]^+^	544.3403	544.3399	−0.7	C_28_H_50_NO_7_P	526.3293 / 184.0735 / 104.1073 / 86.0967	2
55	LPC 16:1	9.54	[M + H]^+^	494.3247	494.3242	−1.0	C_24_H_48_NO_7_P	184.0737 / 104.1075 / 86.0968	2
57	LPE 16:0	9.74	[M-H]^−^	452.2777	452.2762	−3.3	C_21_H_44_NO_7_P	255.2322 / 196.0385 / 140.0124	1
58	LPG 20:5	9.77	[M-H]^−^	529.2566	529.2565	−0.2	C_26_H_43_O_9_P	301.2166 / 257.2267 / 245.0423 / 227.0317 / 171.0050 / 152.9955	2
59	LPE 20:3	9.80	[M-H]^−^	502.2934	502.2935	0.2	C_25_H_46_NO_7_P	305.2468 / 214.0469 / 196.0373 / 140.0133	2
60	LPC 16:0	9.93	[M + H]^+^	496.3403	496.3401	−0.4	C_24_H_50_NO_7_P	478.3288 / 184.0735 / 104.1074 / 86.0967	1
61	LPC 20:3	9.97	[M + H]^+^	546.3560	546.3557	−0.5	C_28_H_52_NO_7_P	528.3456 / 184.0734 / 104.1073 / 86.0969	2
62	LPA 20:5	10.23	[M-H]^−^	455.2199	455.2188	−2.4	C_23_H_37_O_7_P	301.2165 / 171.0040 / 152.9950	2
63	LPC 18:1	10.43	[M + H]^+^	522.3560	522.3546	−2.7	C_26_H_52_NO_7_P	504.3457 / 184.0722 / 104.1073 / 86.0957	2
64	LPG 20:4	10.92	[M-H]^−^	531.2723	531.2718	−0.9	C_26_H_45_O_9_P	303.2321 / 259.2419 / 227.0324 / 171.0032 / 152.9935	2
65	LPC 20:2	11.00	[M + H]^+^	548.3716	548.3705	−2.0	C_28_H_54_NO_7_P	530.3641 / 184.0737 / 104.1071 / 86.0979	2
66	LPG 16:1	11.20	[M-H]^−^	481.2566	481.2563	−0.6	C_22_H_43_O_9_P	253.2164 / 245.0426 /227.0319 / 171.0054 / 152.9955	2
68	LPG 16:0	11.86	[M-H]^−^	483.2723	483.2723	0.0	C_22_H_45_O_9_P	255.2323 / 245.0426 / 227.0311 / 171.0054 / 152.9955	1
69	LPC 20:1	12.44	[M + H]^+^	550.3873	550.3870	−0.5	C_28_H_56_NO_7_P	532.3768 / 184.0740 / 104.1076 / 86.0976	
	
Glycolipids									
56	DGMG 16:0	9.67	[M + Na]^+^	677.3724	677.3722	−0.3	C_31_H_58_O_14_	515.3187	2
67	SQMG 16:0	11.27	[M-H]^−^	555.2839	555.2838	−0.2	C_25_H_48_O_11_S	255.2322 / 225.0068	2
	
Others									
6	Glycerophosphocholine	0.72	[M + H]^+^	258.1106	258.1102	−1.5	C_8_H_20_NO_6_P	184.0733 / 125.0001 / 104.1072	3
7	Isethionic acid	0.73	[M-H]^−^	124.9909	124.9914	4.0	C_2_H_6_O_4_S	106.9806 / 94.9808 / 79.9575	3
8	Glycerophosphoglycerol	0.74	[M-H]^−^	245.0426	245.0431	0.4	C_6_H_15_O_8_P	171.0063 / 152.9956	3
10	Galactosylglycerol	0.83	[M + Na]^+^	277.0899	277.0897	−0.7	C_9_H_18_O_8_	163.0600 / 93.0547	1
43	Phycoerythrobilin	6.87	[M + H]^+^	587.2870	587.2860	−1.7	C_33_H_38_N_4_O_6_	464.2166 / 450.2017	2

MSI levels refer to identification confidence according to the Metabolomics Standards Initiative guidelines. Level 1 indicates identified compounds, confirmed with authentic standards (matching both retention time and mass spectra). Level 2 refers to putatively annotated compounds based on spectral matches to databases or the literature. Level 3 denotes putatively characterized compound classes, assigned based on spectral similarity to known chemical classes. RT, retention time; 5’-CMP, cytidine 5′-monophosphate; 5’-UMP, 5′-uridine monophosphate; 5’-AMP, 5′-adenosine monophosphate, 5’-GMP, 5′-guanosine monophosphate; 5’-IMP, inosine 5′-monophosphate; LPC, lysophosphatidylcholine; LPE, lysophosphatidylethanolamine; LPA, lysophosphatidic acid; LPG, lysophosphatidylglycerol; LPI, lysophosphatidylinositol; DGMG, digalactosylmonoacylglycerol; SQMG, sulfoquinovosylmonoacylglycerol.

Previous studies have reported that dehydrated MAAs are generated during the heating of porphyra-334 and palythine (Nishida et al., 2022; Yoshiki et al., 2009). In this study, peaks corresponding to dehydrated MAAs were also detected in the control samples, suggesting that heat exposure during the drying process may have contributed to their formation. These compounds, which increased with roasting temperature and time, were putatively identified as dehydrated shinorine, dehydrated palythine, dehydrated asterina-330, dehydrated aplysiapalythine B, dehydrated porphyra-334, and dehydrated mycosporine-glycine-alanine (Fig. 1). Among these, dehydrated porphyra-334 was isolated and purified, and its structural assignment was supported by ^1^H NMR analysis (Fig. S3). In contrast, the remaining five dehydrated MAAs were putatively characterized based solely on the MS/MS data obtained in the present study and therefore require further structural confirmation.

**Fig. 1:**
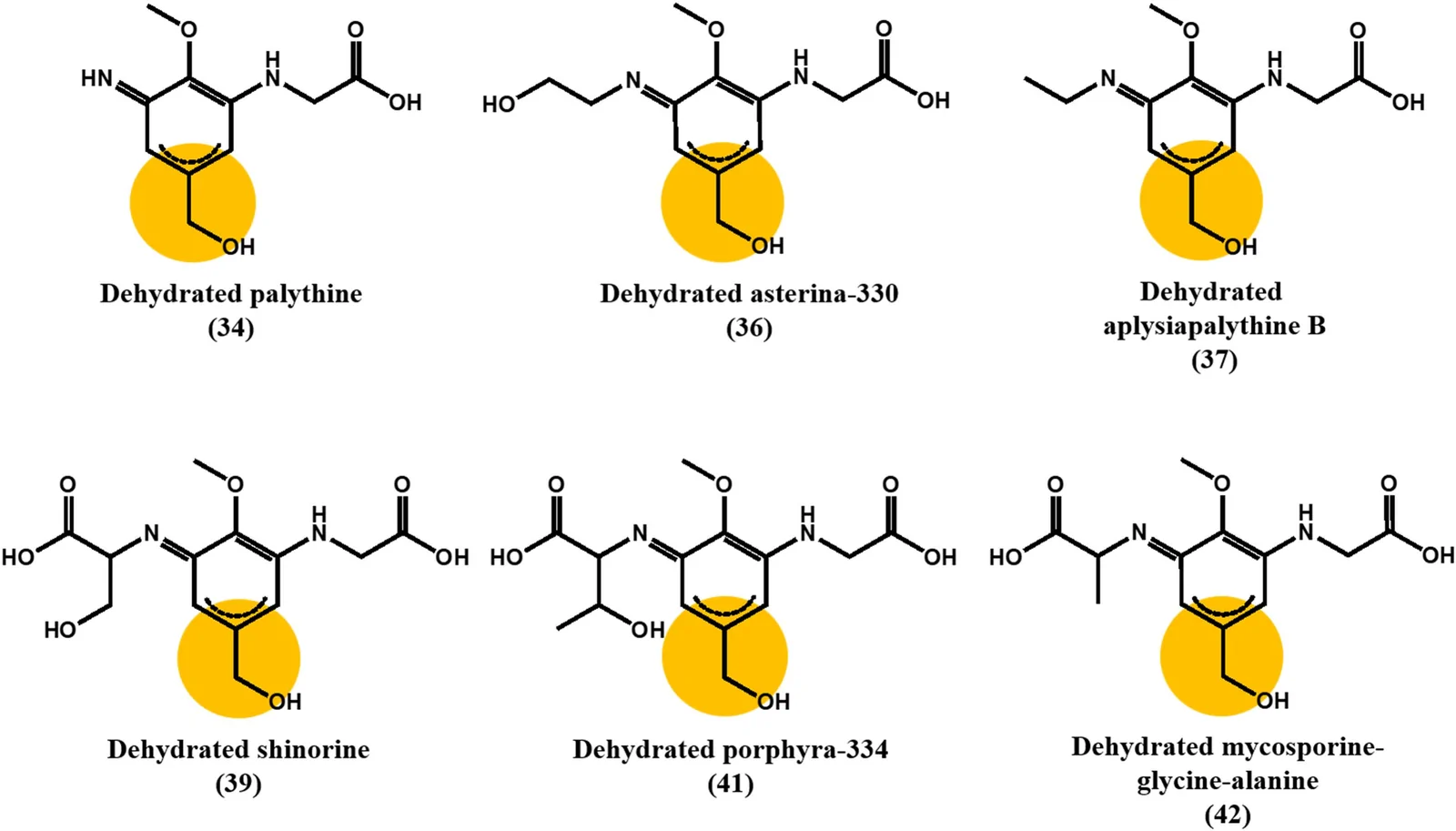
Chemical structures of dehydrated mycosporine-like amino acids tentatively identified using UPLC-QTOF-MS.

The ESI-MS (positive ion mode) spectra of dehydrated MAAs generated by roasting dried laver are shown in Table 1, and the molecular formula of each compound was confirmed by high-resolution mass spectrometry (HRMS) analysis based on the [M + H]^+^ ion. In the MS spectra of peaks 34, 36, 37, 39, 41, and 42, protonated molecular ion peaks [M + H]^+^ were observed at *m/z* 227, 271, 255, 315, 329, and 299, respectively, thereby confirming the molecular weights of the corresponding dehydrated MAAs. The water loss ion [M–H₂O + H]^+^ and the methyl alcohol loss ion [M–CH_2_O + H]^+^ were observed as common fragment ions of dehydrated MAAs. In addition, decarboxylation fragment ions were observed together with dehydration ions [M–H_2_O + H]^+^. The dehydration fragment ion [M–H_2_O + H]^+^ was observed as the most abundant peak for dehydrated palythine, dehydrated aplysiapalythine B, and dehydrated mycosporine-glycine-alanine, whereas the combined dehydration and demethoxylation fragment ion [M–H_2_O–CH_3_OH + H]^+^ was the most abundant peak for dehydrated asterina-330, dehydrated shinorine, and dehydrated porphyra-334. Although the demethylation fragment ion [M–CH_3_ + H]^+^ was reported as a common feature of MAAs (Parailloux et al., 2020), the present study confirmed that fragmentation of dehydrated MAAs occurred predominantly through the [M–H_2_O + H]^+^ ion.

Dehydrated palythine (peak 34) exhibited a protonated molecular ion [M + H]^+^ at *m/z* 227, and the dehydrated fragment ion [M–H_2_O + H]^+^ was identified at *m/z* 209 with a relative abundance of 100% (Fig. 2). This ion produced an *m/z* 181 [M + H–H_2_O–CO]^+^ fragment through a decarboxylation reaction, while subsequent dehydration and dealkylation yielded fragment ions at *m/z* 163 and 151. Additionally, the ion [M–CH_2_O + H]^+^, resulting from the loss of CH_2_O from the parent molecule, was detected at *m/z* 197. The ion [M–CH_3_ + H]^+^, corresponding to the loss of a methyl group, was identified at *m/z* 212, and continuous fragmentations yielded additional ions at *m/z* 194, 168, and 166. Dehydrated aplysiapalythine B (peak 37) and dehydrated mycosporine-glycine-alanine (peak 42) exhibited fragmentation patterns similar to those of dehydrated palythine, with comparable relative intensities (Fig. S4A, B). Dehydrated asterina-330 (peak 36), dehydrated shinorine (peak 39), and dehydrated porphyra-334 (peak 41) exhibited [M + H]^+^ ions at *m/z* 271, 315, and 329, respectively. Although the dehydrated fragment ion [M–H₂O + H]^+^ was commonly detected, the predominant fragment ion was identified as the demethoxylated species [M–H_2_O–CH_3_OH + H]^+^ (Fig. S4C–E). These three compounds exhibited overall similar fragmentation patterns; however, dehydrated shinorine and dehydrated porphyra-334 showed relatively lower intensities of the [M–CH_3_ + H]^+^ ion. In this study, dehydrated MAAs were detected for the first time in roasted *N. yezoensis* using LC-MS. Although HRMS data and observed fragmentation patterns support the proposed structures, future work involving isolation of these compounds and structural validation by NMR spectroscopy is needed to unequivocally confirm their identities. Furthermore, given the diversity of MAAs among seaweed species, additional studies are warranted to clarify the occurrence, structural diversity, and physiological activities of dehydrated MAAs formed during roasting.

**Fig. 2:**
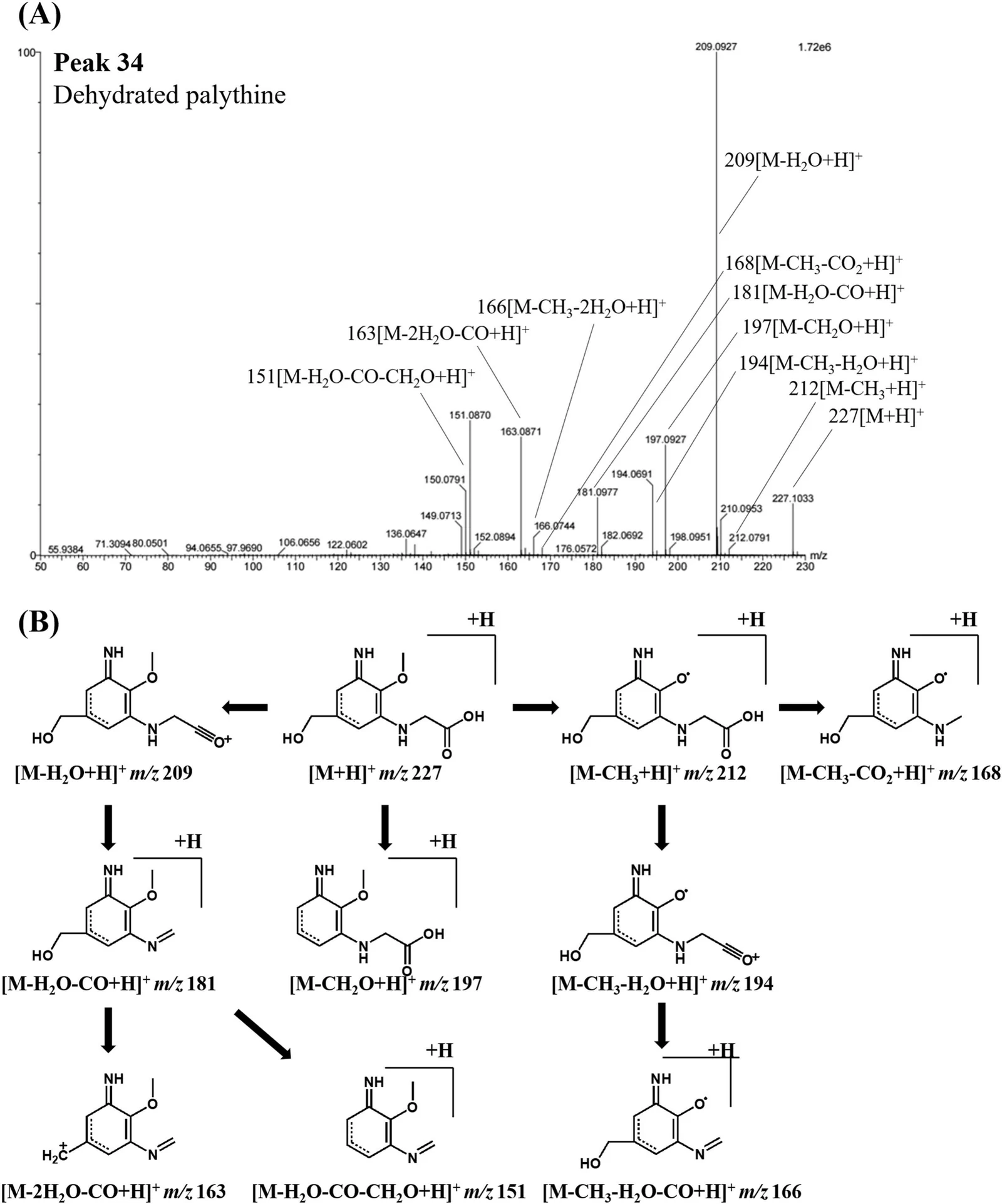
MS/MS (positive ion mode) spectrum (**A**) and fragmentation pathways (**B**) of dehydrated palythine obtained from roasted laver.

### 3.2 Metabolite changes in roasted laver according to roasting temperature and time

PCA was performed on the identified compounds to provide insight into the overall metabolite change and to visualize differences in roasted laver across roasting temperatures and times. The QC samples clustered tightly in the PCA score plot, demonstrating high analytical reproducibility and minimal instrumental drift (Fig. S5A). The median RSD of features in the QC samples was 5.3%, with no missing values detected after filtering (Fig. S5B), confirming the robustness of the metabolite profiling data. Principal components PC1 and PC2 accounted for 70.7% and 18.2% of the total variance, respectively. Along PC1, roasted laver produced at low roasting temperature and short time conditions clustered together, while those produced at high temperature and prolonged roasting time formed a separate group (Fig. 3A). Notably, the PCA loading plot indicated that six dehydrated MAAs (see Section 3.1) were major contributors to the separation along PC1 (Fig. 3B). These results demonstrate that roasting conditions are associated with clear shifts in the metabolite profile of laver.

**Fig. 3:**
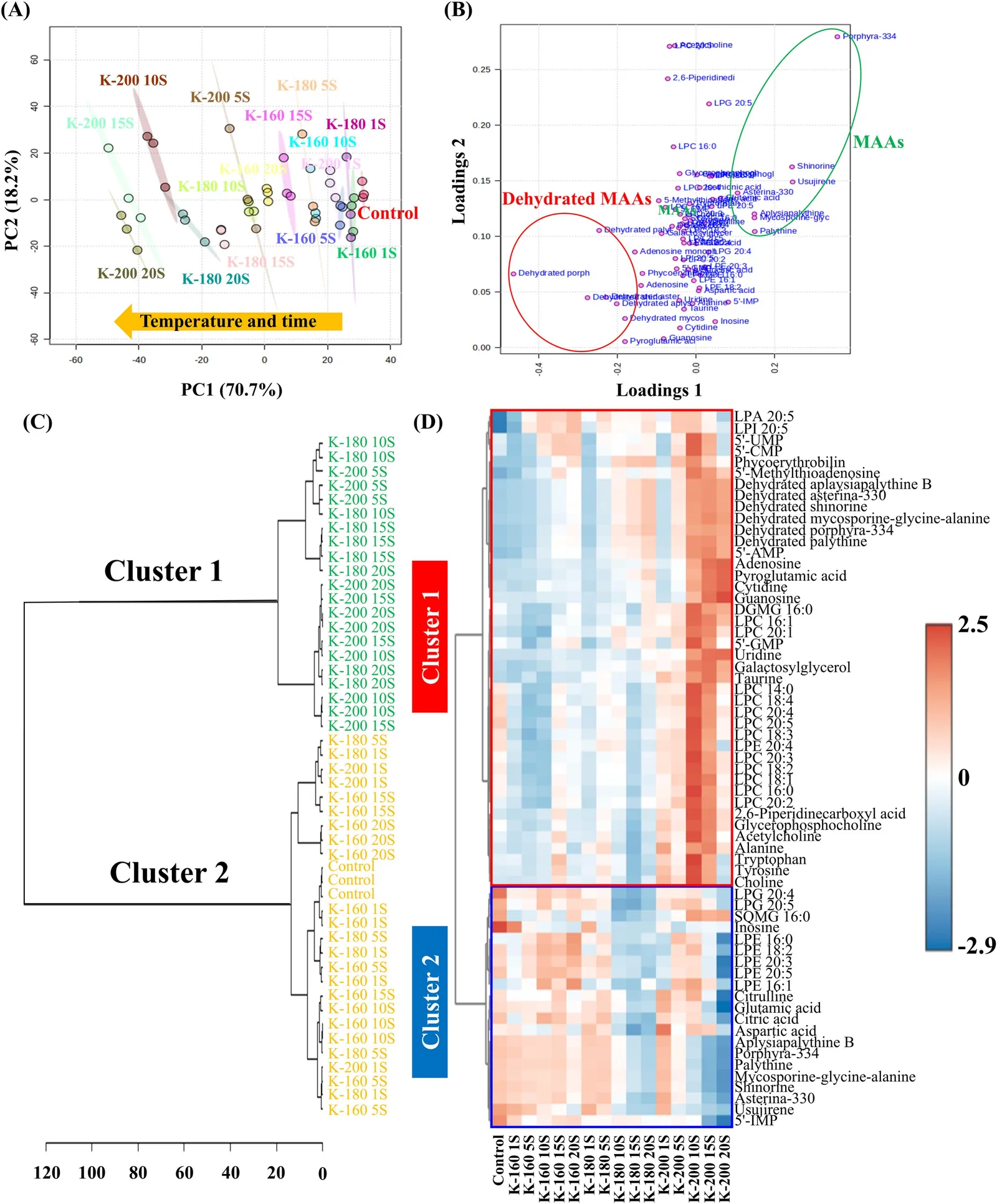
PCA score plot (**A**) and loading plot (**B**), hierarchical clustering dendrogram (**C**), and heatmap (**D**) derived from UPLC-QTOF-MS analysis of roasted laver. Roasting temperatures: 160, 180, and 200 °C; roasting times: 1, 5, 10, 15, and 20 s. LPC, lysophosphatidylcholine; LPE, lysophosphatidylethanolamine; LPA, lysophosphatidic acid; LPG, lysophosphatidylglycerol; LPI, lysophosphatidylinositol; DGMG, digalactosylmonoacylglycerol; SQMG, sulfoquinovosylmonoacylglycerol.

To further clarify the clustering patterns among roasted laver samples, HCA was conducted using Spearman's distance measure and Ward's algorithm (Fig. 3C). As a result, the roasted laver samples were clearly separated into two main clusters in the HCA. Cluster 1 included roasted laver samples produced at 180 °C for 10–20 s and at 200 °C for 5–20 s, whereas cluster 2 included those produced at 160 °C for 1–20 s, at 180 °C for 1–5 s, and at 200 °C for 1 s. These results suggest that metabolite changes within each cluster were similar, indicating that specific combinations of roasting temperature and time produced comparable metabolite profiles. At 160 °C, no significant differences in metabolite composition were observed regardless of roasting time; however, at 180 and 200 °C, the differences became more pronounced with increasing roasting time. In general, high-temperature treatment has been reported to cause losses of nutrients and bioactive components in food (Grzelczyk et al., 2022; Liu et al., 2023; Najafabadi et al., 2023). However, in this study, the compositional changes were relatively minor under 160 °C roasting conditions, likely due to the relatively short roasting time. This suggests that high-temperature treatment exerted a greater impact on the metabolite profile of laver compared to low-temperature treatment.

To further assess the relative contributions of roasting temperature, roasting time, and their interaction to overall variation in metabolites, ASCA was performed (Fig. S6A–C). Both temperature (*p* < 0.005) and time (*p* = 0.005) significantly affected the metabolite profile, whereas their interaction was not significant (*p* = 0.25). Notably, temperature accounted for a larger proportion of total variance than time, indicating it was the dominant factor underlying the clustering patterns observed in PCA and HCA. Additionally, leverage/SPE analysis of the ASCA models identified metabolites that contributed most strongly to temperature- and time-related variation (Fig. S6D–E; Table S2). Variables with high leverage and low SPE were considered influential contributors to the respective factor models. Most of these metabolites were MAAs and dehydrated MAAs—including dehydrated porphyra-334, dehydrated shinorine, shinorine, dehydrated palythine, dehydrated asterina-330, dehydrated aplysiapalythine B, and dehydrated mycosporine-glycine-alanine—indicating that these compounds were major contributors to roasting-associated variation in the metabolite profile.

At the individual metabolite level, two-way ANOVA identified 63 metabolites significantly affected by roasting temperature, time, and/or their interaction (*q* < 0.05). A Venn diagram summarizing these effects is shown in Fig. S7, and the corresponding metabolites are listed in Table S3. Although the interaction effect was not significant at the global ASCA level, several individual metabolites exhibited significant temperature × time interaction effects. The changes in these 63 significantly altered metabolites among the 69 identified in roasted laver were visualized using a heatmap (Fig. 3D). These metabolites were separated into two distinct clusters based on their relative abundances. Cluster 1 metabolites were primarily composed of nucleic acid-related compounds, along with lysophosphatidylcholines (LPC), dehydrated MAAs, digalactosylmonoacylglycerol (DGMG) 16:0, lysophosphatidic acid (LPA) 20:5, lysophosphatidylinositol (LPI) 20:5, galactosylglycerol, glycerophosphocholine, and phycoerythrobilin. Their contents generally increased with higher roasting temperature and longer roasting time, with the highest levels generally observed at 200 °C. In contrast, cluster 2 metabolites, including lysophosphatidylethanolamine (LPE), lysophosphatidylglycerol (LPG), MAAs, and sulfoquinovosylmonoacylglycerol (SQMG) 16:0, decreased with increasing roasting temperature and time. Amino acids and their derivatives were distributed across both clusters, although most were included in cluster 1. The contrasting cluster patterns may reflect multiple thermal transformation pathways. The increase in nucleic acid-related compounds in cluster 1 may be associated with the thermal degradation of nucleic acids (Xu et al., 2022), whereas the accumulation of lysophospholipids may reflect the conversion of membrane phospholipids during roasting (Mroz et al., 2024). The simultaneous increase in dehydrated MAAs and decrease in MAAs is consistent with heat-induced dehydration. The decrease in other polar lipids in cluster 2 may indicate their thermal conversion or degradation under more intense roasting conditions. The distribution of amino acids across both clusters may reflect differences in their thermal stability and participation in reactions such as Strecker degradation and the Maillard reaction (Pei et al., 2014).

### 3.3 Metabolite differences in roasted laver according to roasting temperature and time

#### 3.3.1 Amino acids and their derivatives

Amino acids and their derivatives are not only the fundamental constituents of proteins but also serve as precursors for a wide range of metabolites. They play critical roles in maintaining homeostasis throughout the life cycle of organisms, including cellular function, growth, and development (Huang, Peng and Yan, 2021). In addition, amino acids are recognized as key contributors to food flavor, together with nucleic acid-related compounds, peptides, organic acids, free sugars, and carbonyl compounds (Brand & Bryant, 1994). LC-MS metabolite analysis identified a total of 11 amino acids and their derivatives, and their content changes under different roasting conditions are presented in Fig. 4A. The contents of total amino acids and their derivatives varied by 0.92–1.25-fold compared with dried unroasted laver (control). Although no significant differences were observed in most treatment groups, the highest levels were detected in laver roasted at 200 °C for 10 s. Analysis of individual amino acids and their derivatives revealed that the contents of taurine (0.99–1.14-fold), alanine (0.93–1.17-fold), choline (0.95–1.19-fold), 2,6-piperidinedicarboxylic acid (0.91–1.43-fold), tyrosine (0.87–1.41-fold), tryptophan (0.86–1.39-fold), and acetylcholine (0.92–1.19-fold) were maintained or slightly increased with higher roasting temperature. In particular, pyroglutamic acid, a cyclized derivative of L-glutamic acid, significantly increased with roasting temperature and time, reaching up to 23.54-fold higher levels in laver roasted at 200 °C for 20 s compared to the unroasted control. A previous study reported that pyroglutamic acid is readily formed through the dehydration–cyclization of glutamic acid at 140–220 °C (Wu et al., 2009).

**Fig. 4:**
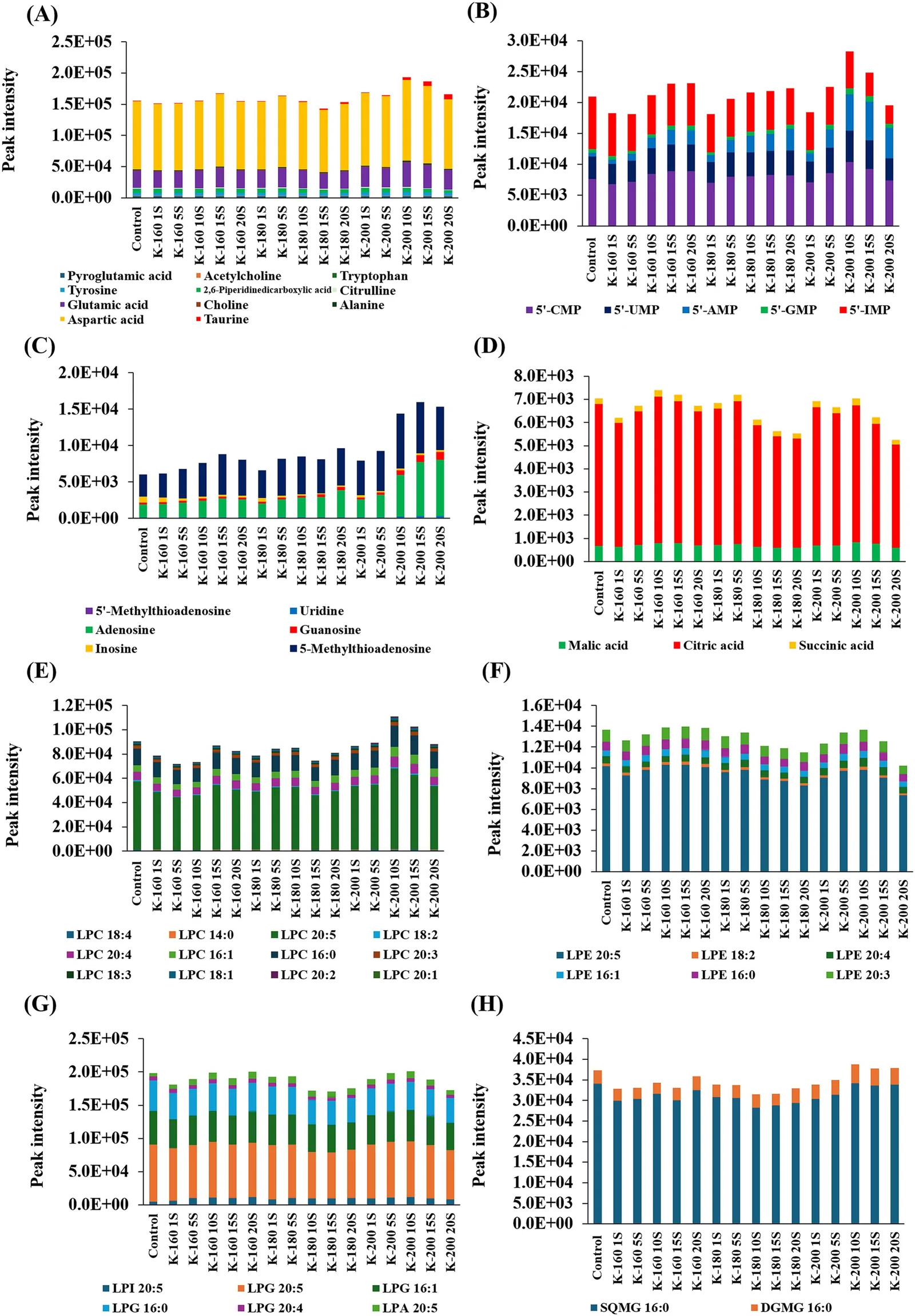
Changes in the contents of amino acids (**A**), 5′-nucleotides (**B**), nucleosides (**C**), organic acids (**D**), and lipids (**E**–**H**) (LPC, lysophosphatidylcholine; LPE, lysophosphatidylethanolamine; LPG, lysophosphatidylglycerol; LPA, lysophosphatidic acid; LPI, lysophosphatidylinositol; SQMG, sulfoquinovosyl monoacylglycerol; DGMG, digalactosyl monoacylglycerol) in roasted laver. Roasting temperatures: 160, 180, and 200 °C; roasting times: 1, 5, 10, 15, and 20 s. Values are presented as mean ± standard error (*n* = 3).

Consistent with the formation of pyroglutamic acid, glutamic acid (0.57–1.09-fold) showed the lowest level under the roasting condition of 200 °C for 20 s. These reciprocal changes suggest that the marked accumulation of pyroglutamic acid resulted, at least in part, from the heat-induced dehydration and cyclization of glutamic acid. Accordingly, the accumulation of pyroglutamic acid reflects the roasting-induced transformation of glutamic acid under relatively intense heating conditions. Similarly, aspartic acid (0.89–1.09-fold) and citrulline (0.66–1.09-fold) also decreased with increasing roasting temperature and time. These changes may be attributed to the Strecker degradation of amino acids or to the Maillard reaction between amino acids and reducing sugars (Pei et al., 2014). Glutamic acid, alanine, and aspartic acid are key amino acids contributing to the characteristic flavor of laver. Variations in their contents induced by heat treatment may therefore influence the flavor profile of roasted laver. These findings highlight the importance of roasting conditions in determining amino acid retention, thermal transformation, and the flavor-related chemical composition of roasted laver.

#### 3.3.2 Nucleic acid-related compounds

LC-MS analysis detected five 5′-nucleotides and six nucleosides in laver. As shown in Fig. 4B, the total contents of 5′-nucleotides varied between 0.86- and 1.35-fold during the roasting process, and they generally increased with longer roasting times at each temperature. In particular, compared with dried unroasted laver, the 5′-nucleotide content was highest in laver roasted at 200 °C for 10 s. For nucleosides (Fig. 4C), the total contents varied from 1.03- to 2.65-fold depending on the roasting conditions. No significant changes were observed at 160 and 180 °C; however, at 200 °C, nucleoside contents increased markedly with longer roasting times. This suggests that DNA or RNA present in laver may be decomposed during high-temperature treatment, leading to the conversion into various types of 5′-nucleotides or nucleosides (Xu et al., 2022). Additionally, in samples roasted at 200 °C for 20 s, the contents of 5′-nucleotides decreased, whereas those of nucleosides simultaneously increased. This suggests that 5′-nucleotides may have undergone further thermal decomposition at high temperatures, resulting in their conversion to nucleosides.

Analysis of individual nucleic acid-related compounds revealed that cytidine 5′-monophosphate (5’-CMP) and inosine 5′-monophosphate (5’-IMP) were the most abundant, consistent with a previous study that examined the metabolite profile of *N. yezoensis* under different drying methods (Yan et al., 2025). The change patterns of individual compounds were consistent with those of the total 5′-nucleotide and nucleoside contents. Specifically, 5′-adenosine monophosphate (5’-AMP, 1.18–10.69-fold), 5’-CMP (0.90–1.36-fold), 5′-guanosine monophosphate (5’-GMP, 0.70–1.59-fold), 5′-uridine monophosphate (5’-UMP, 0.88–1.38-fold), adenosine (1.02–4.34-fold), cytidine (0.86–3.34-fold), guanosine (0.89–4.08-fold), uridine (0.98–2.14-fold), and 5′-methylthioadenosine (1.10–2.50-fold) all tended to increase with higher roasting temperature and longer roasting time. In contrast, the contents of 5’-IMP (0.35–0.82-fold) and inosine (0.21–0.79-fold) decreased with increasing heat treatment intensity. A similar trend was reported by Pei et al. (2014); the levels of 5’-GMP and 5’-AMP increased during heat treatment, whereas 5’-IMP decreased. Because 5′-nucleotides possess weak chemical bonds such as glycosidic or ester linkages, they can be decomposed by heat treatment (Matoba et al., 1988). This suggests that 5’-IMP and inosine are particularly sensitive to high temperatures or prolonged heat exposure, and that they readily degrade into ribose. Meanwhile, 5′-nucleotides such as 5’-AMP, 5’-GMP, 5’-IMP, and 5’-UMP are known to play a crucial role in the formation of umami taste, together with aspartic acid and glutamic acid among amino acids (Dermiki et al., 2013). Among the samples examined in the present study, laver roasted at 200 °C for 10 s exhibited the highest contents of total amino acids and total 5′-nucleotides, suggesting that this roasting condition may be favorable for achieving higher levels of taste-related compounds.

#### 3.3.3 Organic acids

In a previous study, seven organic acids, including oxalic acid, citric acid, tartaric acid, malic acid, succinic acid, lactic acid, and acetic acid, were reported in *N. yezoensis* (Park et al., 2014). In the present study, three organic acids —citric acid, malic acid, and succinic acid—were detected in laver (Fig. 4D). The total organic acid content varied from 0.75- to 1.05-fold compared with the control sample, depending on roasting temperature and time, and generally decreased with increasing roasting intensity. This trend is consistent with previous findings showing that the organic acid content of jujubes decreased after heating at 80 and 95 °C (Najafabadi et al., 2023). In chestnuts, roasting, boiling, and frying significantly reduced the organic acid content, with fried chestnuts showing the lowest levels (Ribeiro et al., 2007). This may be attributed to decarboxylation induced by heat treatment (Pei et al., 2014).

Meanwhile, changes in individual organic acids exhibited trends similar to those of the total organic acids. Organic acids primarily function to increase food acidity and impart a characteristic sour flavor. Succinic acid has been reported to enhance the intensity of umami and saltiness (Du et al., 2025). Citric acid and malic acid are not only important nutrients, particularly in fruits, but also make significant contributions to flavor (Jia et al., 2023).

#### 3.3.4 Lipids

Roasting temperature and time are critical factors influencing lipid oxidation and hydrolysis. During the roasting process, lipids are susceptible to various heat-induced reactions that can affect the flavor and nutritional properties of foods. Phospholipids, a class of polar lipids and major constituents of cell membranes, can be converted into lysophospholipids during heat treatment as free fatty acids are released from their structures (Mroz et al., 2024). Lysophospholipids are classified into LPA, LPC, LPE, lysophosphatidylserine (LPS), LPI, and LPG, depending on the polar group attached to the sn-3 position of the glycerol backbone. These compounds have been reported to act as signaling molecules in response to various abiotic stresses in plants, including salinity, temperature, and nitrogen or phosphorus deficiency (Lopes et al., 2024). Red algae are known to contain higher levels of phospholipids than green and brown algae (Lopes et al., 2021), which may also influence the lipid metabolite profile of laver.

As shown in Fig. 4E, the total LPC content varied from 0.79- to 1.23-fold with increasing roasting temperature and time, and individual LPC species exhibited similar trends. In particular, the LPC content reached its highest level in samples roasted at 200 °C for 10 s. In addition, the contents of LPA 20:5 and LPI 20:5 increased by 1.30–2.08- and 1.32–2.47-fold, respectively, in response to roasting treatment. This suggests that during the roasting process, phosphatidic acid and phosphatidylinositol underwent the most active hydrolysis reactions, yielding LPA and LPI, respectively. In contrast, LPE and LPG showed decreasing trends, ranging from 0.84 to 1.02- and 0.81–0.95-fold, respectively (Fig. 4F, G). Different polar groups of phospholipids can participate in hydrogen bonding, which may influence their thermal stability and thereby affect the efficiency of conversion to lysophospholipids. Meanwhile, two types of glycolipids were also identified in roasted laver, with SQMG 16:0 ranging from 0.83 to 1.01-fold and DGMG 16:0 ranging from 0.83 to 1.40-fold (Fig. 4H). These different responses may reflect structure-dependent differences in the thermal stability of glycolipids. Although laver contains relatively low levels of polar lipids, it is an important dietary source of n-3 PUFAs, particularly EPA, highlighting the relevance of understanding lipid transformations during roasting (Lopes et al., 2021).

#### 3.3.5 Mycosporine-like amino acids (MAAs)

MAAs are well known to be abundant in laver. LC-MS-based metabolite analysis of roasted laver revealed seven types of MAAs and six types of dehydrated MAAs. Multivariate analysis identified these compounds as the primary contributors to the differences observed among roasted laver groups under different roasting conditions. The changes in six dehydrated MAAs in roasted laver are shown in Fig. 5, whereas those in seven MAAs are presented in Fig. S8. The effects of temperature, time, and their interaction on these compounds are summarized in Table S4. As roasting temperature and time increased, the contents of MAAs decreased to as low as 0.06-fold for shinorine, 0.14-fold for palythine, 0.28-fold for asterina-330, 0.07-fold for porphyra-334, 0.04-fold for mycosporine-glycine-alanine, 0.07-fold for aplysiapalythine B, and 0.07-fold for usujirene, with the most pronounced reductions observed at 200 °C. In contrast, dehydrated MAAs generated from MAAs during roasting increased by up to 28.62-fold for dehydrated shinorine, 8.40-fold for dehydrated palythine, 40.67-fold for dehydrated asterina-330, 13.14-fold for dehydrated porphyra-334, 62.10-fold for dehydrated mycosporine-glycine-alanine, and 27.60-fold for dehydrated aplysiapalythine B. However, after increasing at shorter roasting times, their contents began to decrease when the roasting time reached 20 s at 200 °C.

**Fig. 5:**
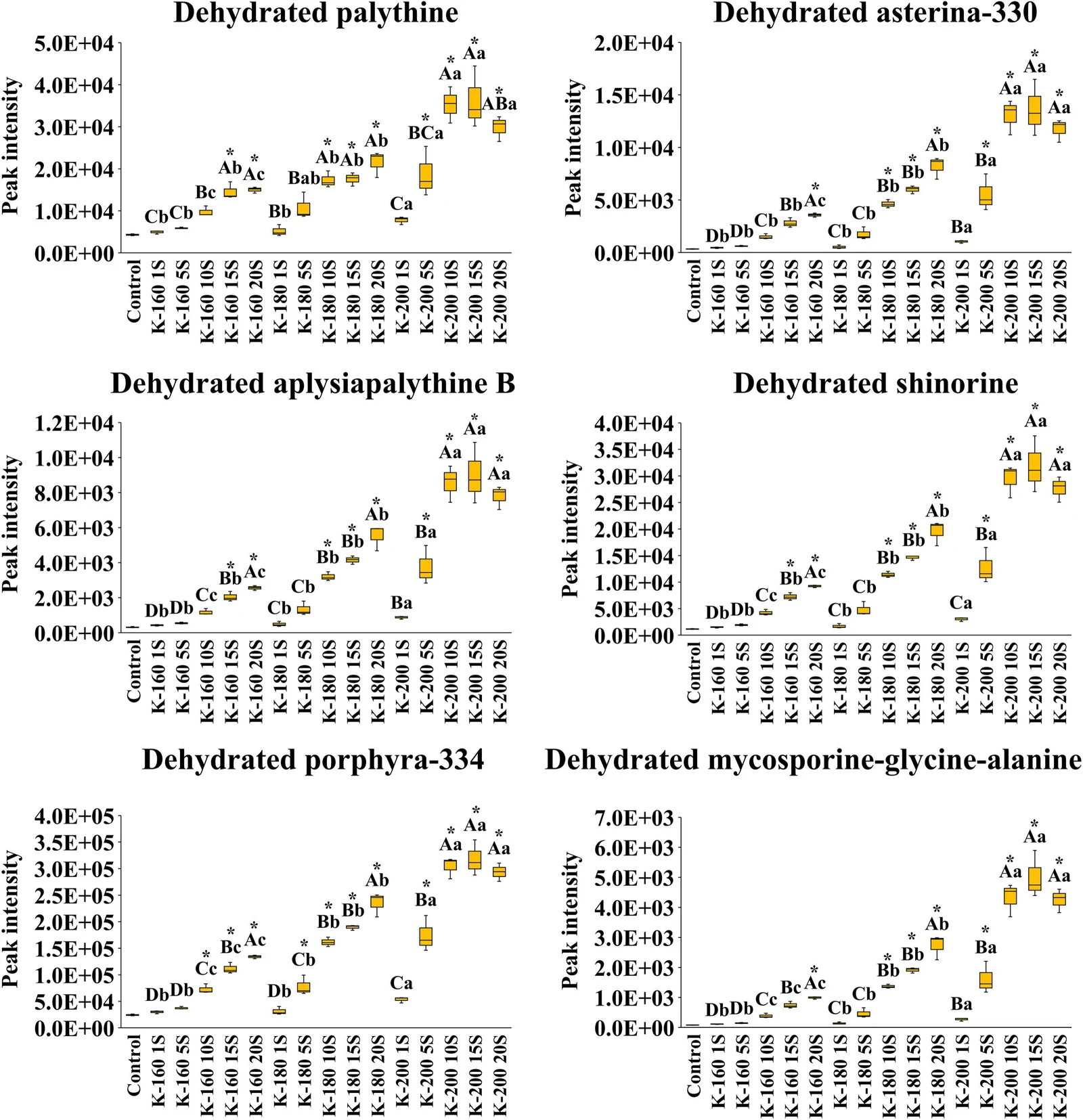
Changes in the contents of six dehydrated MAAs in roasted laver. Roasting temperatures: 160, 180, and 200 °C; roasting times: 1, 5, 10, 15, and 20 s. Values are presented as mean ± standard error (*n* = 3). Different uppercase letters (A–D) within the same roasting temperature indicate significant differences among roasting times (*p* < 0.05); different lowercase letters (a–c) within the same roasting time indicate significant differences among roasting temperatures (*p* < 0.05), according to Tukey's multiple comparison test. Asterisks (*) indicate significant differences between each roasted treatment and the unroasted control according to Dunnett's test (*p* < 0.05). MAA, mycosporine-like amino acid.

Dehydration induced by heating does not occur in all MAAs. The stability of MAAs to heat-induced dehydration depends on their specific chemical structure. Some MAAs exhibit very low thermal stability. Mycosporine-glycine has been reported to be converted into a β-diketone (6-deoxygadusol) and glycine when heated in water at 80 °C for 3 h (Carreto & Carignan, 2011). Shinorine methyl ester (M-333) and the complex MAA M335/360 have been reported to undergo hydrolysis, accompanied by an increase in shinorine, when heated in 25% aqueous methanol at 45 °C for 2 h (Carreto et al., 2005). Furthermore, usujirene has been reported to undergo hydrolysis to palythine, which is subsequently converted into dehydrated palythine through dehydration (Nishida et al., 2022). In this study, the MS spectrum peak at *m/z* 267 (C_13_H_18_N_2_O_4_), corresponding to dehydrated usujirene, was not detected, and the abundance of usujirene decreased significantly with increasing roasting temperature and time. These observations suggest that usujirene may undergo thermal transformation through pathways other than the formation of the proposed dehydrated derivative under the conditions examined.

To date, overseas exports of laver have mainly focused on dried products; however, with the active development of various processed laver foods such as laver snacks, export items have recently begun to diversify (Choi et al., 2020). Laver snack products are typically manufactured by short-term high-temperature heating, generally above 100 °C. A representative example is *gimbu-gak*, a type of laver snack produced by coating laver with glutinous rice paste, drying it, and subsequently frying it in oil. The findings of this study provide fundamental information for understanding and modulating the retention and thermal transformation of MAAs during roasted laver production under comparable short-term, high-temperature conditions.

### 3.4 Antioxidant activity of roasted laver under different roasting temperatures

LC-MS-based metabolomic analysis revealed that the contents of major metabolites in roasted laver—including amino acids and their derivatives, organic acids, MAAs, nucleic acid-related compounds, phospholipids, glycolipids, and dehydrated MAAs—varied with roasting temperature and time. Dehydrated MAAs have been reported as major factors influencing antioxidant activity in previous studies (Nishida et al., 2022; Yoshiki et al., 2009). Therefore, to investigate changes in the antioxidant activity of roasted laver under different roasting conditions, ABTS^+^ radical scavenging activity and FRAP were evaluated. As shown in Fig. 6A, compared to dried unroasted laver, ABTS^+^ radical scavenging activity gradually increased by 1.18–4.53-fold, and FRAP increased by 1.06–4.18-fold as roasting temperature and time increased. In particular, the ABTS^+^ radical scavenging activity of laver roasted for 200 °C was significantly higher than that of dried unroasted laver; however, no significant differences were observed among samples roasted at 200 °C for 10–20 s. In contrast, FRAP gradually increased with longer roasting time, regardless of roasting temperature (Fig. 6B).

**Fig. 6:**
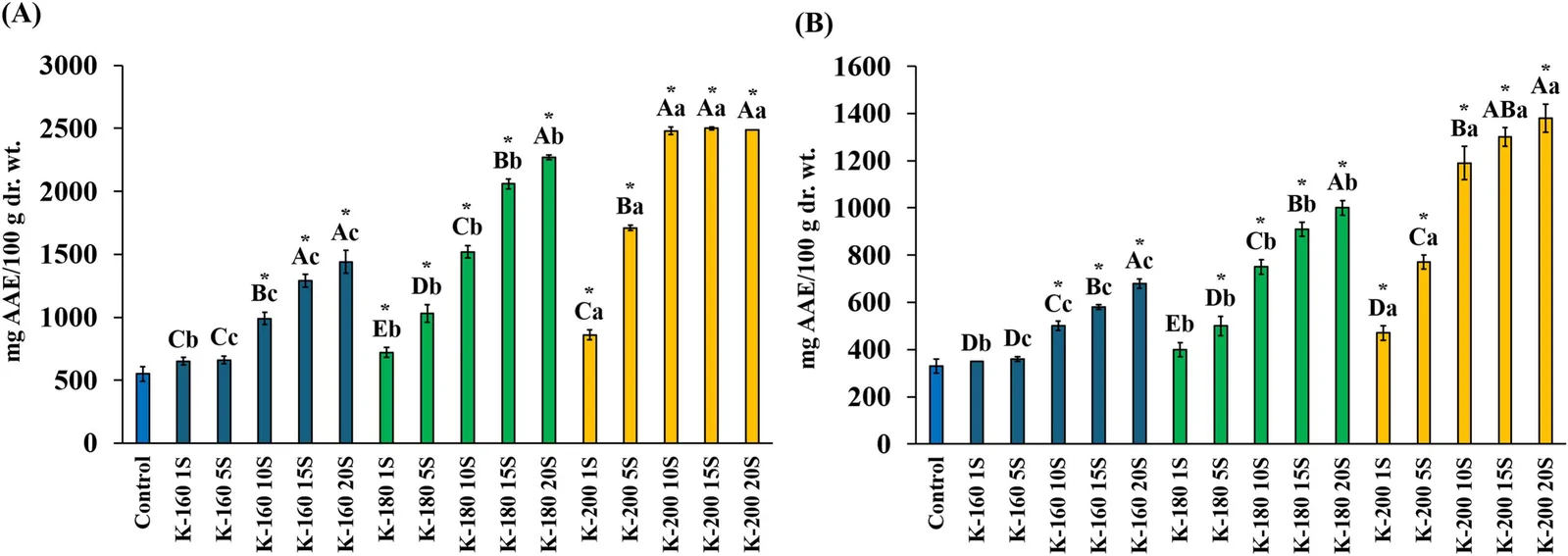
ABTS^+^ radical scavenging activity (**A**) and ferric reducing antioxidant power (**B**) of roasted laver samples. Values are presented as mean ± standard deviation (*n* = 3). Roasting temperatures: 160, 180, and 200 °C; roasting times: 1, 5, 10, 15, and 20 s. Different uppercase letters (A–E) within the same roasting temperature indicate significant differences among roasting times (*p* < 0.05); different lowercase letters (a–c) within the same roasting time indicate significant differences among roasting temperatures (*p* < 0.05), according to Tukey's multiple comparison test. Asterisks (*) indicate significant differences between each roasted treatment and the unroasted control according to Dunnett's test (*p* < 0.05).

In a previous study, the antioxidant activity of heated dulse (*P. palmata*) extracts was found to decrease slightly at 60–105 °C but increased at 120 °C (Nishida et al., 2022). This suggests that at relatively low temperatures, antioxidant activity may be reduced due to the degradation of heat-labile compounds, whereas at higher temperatures, heat may promote the formation of substances with strong antioxidant activity. In the present study, an overall increase in antioxidant activity was observed when roasting was performed at temperatures above 160 °C. In particular, LC-MS metabolite analysis showed that the contents of dehydrated MAAs in laver remained largely unchanged after roasting at 200 °C for more than 10 s, a trend consistent with that observed for ABTS^+^ radical scavenging activity.

### 3.5 Correlation between metabolites and antioxidant activity

To comprehensively assess the drivers of antioxidant activity, Pearson's correlation analysis was performed between the 69 identified metabolites and antioxidant activity (Table S5). As shown in Table 2, MAAs (shinorine, palythine, asterina-330, aplysiapalythine B, porphyra-334, and mycosporine-glycine-alanine) present in dried unroasted laver exhibited strong negative correlations with ABTS^+^ radical scavenging activity (*r* = −0.93 to −0.81) and FRAP (*r* = −0.94 to −0.81). In contrast, six types of dehydrated MAAs, including dehydrated shinorine and dehydrated porphyra-334, generated during the roasting process exhibited strong positive correlations with ABTS^+^ radical scavenging activity (*r* = 0.93–0.98) and FRAP (*r* = 0.93–0.98). These contrasting correlation patterns suggest that the conversion of MAAs into dehydrated forms during roasting may be closely associated with the increased antioxidant activity observed in roasted laver. Previous studies have reported that dehydrated porphyra-334 and dehydrated palythine possess resonance double-bond structures, such as those between C-4 and C-5 or C-5 and C-6, which may enhance electron delocalization and thereby increase antioxidant capacity (Nishida et al., 2022; Yoshiki et al., 2009). Accordingly, the accumulation of dehydrated MAAs during roasting may partly explain the observed increases in ABTS^+^ radical scavenging activity and FRAP values. Further structural confirmation for the four tentatively identified compounds is required before this mechanism can be generalized to all six dehydrated MAAs.

**Table 2:** Pearson's correlation between antioxidant activity and MAAs detected in roasted laver.

Class	Compounds	ABTS^+^	FRAP
MAAs	Shinorine	−0.93^⁎⁎⁎^	−0.94^⁎⁎⁎^
	Palythine	−0.91^⁎⁎⁎^	−0.93^⁎⁎⁎^
	Asteria-330	−0.81^⁎⁎⁎^	−0.81^⁎⁎⁎^
	Porphyra-334	−0.91^⁎⁎⁎^	−0.92^⁎⁎⁎^
	Mycosporine-glycine-alanine	−0.89^⁎⁎⁎^	−0.90^⁎⁎⁎^
	Aplysiapalythine B	−0.89^⁎⁎⁎^	−0.90^⁎⁎⁎^
	Usujirene	−0.89^⁎⁎⁎^	−0.84^⁎⁎⁎^
	Dehydrated palythine	0.93^⁎⁎⁎^	0.93^⁎⁎⁎^
Dehydrated MAAs	Dehydrated asteria-330	0.94^⁎⁎⁎^	0.96^⁎⁎⁎^
	Dehydrated aplysiapalythine B	0.95^⁎⁎⁎^	0.96^⁎⁎⁎^
	Dehydrated shinorine	0.95^⁎⁎⁎^	0.97^⁎⁎⁎^
	Dehydrated porphyra-334	0.98^⁎⁎⁎^	0.98^⁎⁎⁎^
	Dehydrated mycosporine-glycine-alanine	0.93^⁎⁎⁎^	0.96^⁎⁎⁎^

MAA, mycosporine-like amino acid; *n* = 48; *p*-values were corrected for multiple comparison using the Benjamini–Hochberg FDR method. ^∗∗∗^ indicates FDR-adjusted *q* < 0.001.

Nevertheless, the elevated antioxidant activity of roasted laver should not be attributed exclusively to dehydrated MAAs. Roasting also induced broader metabolic changes beyond the MAA group, and several other metabolites—including 5’-AMP, pyroglutamic acid, adenosine, phycoerythrobilin, cytidine, guanosine, galactosylglycerol, 5′-methylthioadenosine, and 5’-GMP—were significantly associated with antioxidant activity (*r* > 0.70). Some of these compounds are known to possess inherent antioxidant properties or participate in antioxidant-related mechanisms, and their potential contributions cannot be excluded based on the present results (Asadullina et al., 2010; Bliska et al., 2018; Jansen et al., 2020; Yabuta et al., 2010).

Additionally, ABTS^+^ radical scavenging activity and FRAP are chemical assays that reflect overall radical scavenging or reducing capacity rather than compound-specific or biological antioxidant mechanisms, and they may also respond to roasting-derived compounds outside the metabolite classes detected here (Rumpf et al., 2023). Laver contains not only low-molecular-weight compounds like MAAs but also high-molecular-weight components such as polysaccharides and proteins that were not detected in the present LC-MS–based profiling (Cho & Rhee, 2019; Kim et al., 2025). Roasting can induce denaturation, fragmentation, and polymerization of these matrix components, particularly through Maillard-type reactions, generating antioxidant-active compounds such as melanoidins (Ali et al., 2025; Ding et al., 2024).

This interpretation is consistent with previous findings in laver roasting, in which the browning index increased continuously during both pan roasting and air-fryer treatment, but a consistent increase in antioxidant activity was observed only in pan-roasted laver. Moreover, the reported correlation between browning index and antioxidant activity was relatively low (*r* = 0.255–0.361), suggesting that browning alone may not fully account for the antioxidant changes (Lee & Lee, 2024).

Taken together, our results indicate that dehydrated MAAs are significant contributors to the enhanced antioxidant capacity of roasted laver, but their role should be viewed as part of a broader network of roasting-induced chemical changes rather than the sole factor. From an application standpoint, roasting conditions that promote the formation of dehydrated MAAs could be relevant for improving antioxidant-related quality in laver. Further research is needed to clarify the relative contributions of dehydrated MAAs and other roasting-induced compounds to overall antioxidant activity.

## 4 Conclusion

In this study, UPLC-QTOF-MS–based metabolomic analysis was employed to characterize compositional changes in *N. yezoensis* subjected to different roasting temperatures and times. Sixty-nine major metabolites, including MAAs and dehydrated MAAs, were identified, and multivariate analysis revealed distinct clustering of roasted laver samples according to roasting conditions. Roasting induced substantial changes in metabolite profiles, including decreases in MAAs and increases in dehydrated MAAs, as well as broader changes in amino acids, their derivatives, organic acids, nucleotides, nucleosides, and phospholipids. Antioxidant activity, as assessed by ABTS^+^ radical scavenging activity and FRAP, generally increased with roasting intensity. Pearson's correlation analysis suggested that roasting-induced metabolite changes, particularly the accumulation of dehydrated MAAs, were associated with enhanced antioxidant activity. These findings provide insights into the relationships between metabolite changes and antioxidant activity during roasting and offer a biochemical basis for future studies aimed at controlling roasting-induced compositional changes in laver.

## CRediT authorship contribution statement

**Si-Hun Song:** Writing – original draft, Visualization, Validation, Investigation, Formal analysis, Data curation, Conceptualization. **Seung-Hun Chae:** Writing – review & editing, Formal analysis. **Seung-Sik Cho:** Writing – review & editing, Supervision. **Hyung Gyun Kim:** Project administration, Funding acquisition. **Jeong-Yong Cho:** Supervision, Methodology, Conceptualization.

## Declaration of competing interest

The authors declare that they have no known competing financial interests or personal relationships that could have appeared to influence the work reported in this paper.

## Data Availability

The data that support the findings of this study are available in the Supporting Information of this article.
