# Supplementary material for: Roasting-induced changes in the metabolite profile of laver (Neopyropia yezoensis): Focus on mycosporine-like amino acids and antioxidant activity
Source: Food Chem X. 2026 Sep 19;39:104450. doi: 10.1016/j.fochx.2026.104450 (PMC13628688; doi:10.1016/j.fochx.2026.104450)
Supplement: Supplementary file 1 [file mmc1.docx]

**Supplementary Information**

**Roasting-induced changes in the metabolite profile of laver (*Neopyropia yezoensis*): Focus on mycosporine-like amino acids and antioxidant activity**

Si-Hun Song^a^, Seung-Hun Chae^b^, Seung-Sik Cho^c^, Hyung Gyun Kim^d^,

Jeong-Yong Cho^a,^**^*^**

**Supplementary Information**

**Purification and isolation of dehydrated porphyra-334 from roasted laver**

Roasted laver (500 g) was extracted with ethanol (10 L) at 25 °C for 24 h. The extracts were filtered through Whatman No. 2 filter paper (Whatman International Ltd., Maidstone, England) and concentrated *in vacuo*. The ethanolic extract (14.53 g) was subjected to medium-pressure liquid chromatography (MPLC; Isolera, Biotage, Uppsala, Sweden) equipped with an ODS column (Sfar C_18_ Duo 240 g, Biotage). The compounds were purified using gradient elution of 100% H_2_O (0–5 min) to 30% acetonitrile (MeCN, 5–20 min) at a flow rate of 50 mL/min. The dehydrated porphyra-334 rich fraction (364.3 mg, 10–17% MeCN) was further purified by MPLC using a silica gel column (Sfar silica 100 g, Biotage) and step-wise elution of CHCl_3_/MeOH/H_2_O = 16:4:1, 12:8:1, and 8:12:1 v/v (10 min each, flow rate 25 mL/min) to isolate dehydrated porphyra-334 (*t_R_* 31.8 min, 23.5 mg).

**Identification of dehydrated porphyra-334 from the roasted laver**

The chemical structure of the isolated dehydrated porphyra-334 was elucidated using MS and NMR analyses. The ^1^H nuclear magnetic resonance (NMR) spectrum of the isolated compound was obtained in deuterium oxide (D_2_O) using a Unity INOVA 500 spectrometer (Varian, Walnut Creek, CA, USA) at the Korean Basic Science Institute, Gwangju Center. The ^1^H-NMR spectrum (Fig. S3) showed proton signals corresponding to a cyclohexanone ring, including a methine proton at δ 6.13–6.19, a methylene proton at δ 2.09 (H-4 or H-6), an oxygenated methylene proton at δ 3.83 (H-7), and a methoxy proton at δ 3.37 (H-8). The proton signals corresponding to glycine (methylene at δ 4.48, H-9) and threonine [an oxygenated methine at δ 4.15 (H-13), a methyl group at δ 1.33 (H-14), and a nitrogenated methylene at δ 3.75 (H-12)] were additionally observed. The ^1^H-NMR data were consistent with those of dehydrated porphyra-334 reported by Yoshiki et al. (2009).

**Table S1.** Roasted laver sample names and roasting conditions.

| No. | Sample | Roasting temperatures (℃) | Roasting time (s) |
| --- | --- | --- | --- |
| 1 | Control | 0 | 0 |
| 2 | K-160 1S | 160 | 1 |
| 3 | K-160 5S | 160 | 5 |
| 4 | K-160 10S | 160 | 10 |
| 5 | K-160 15S | 160 | 15 |
| 6 | K-160 20S | 160 | 20 |
| 7 | K-180 1S | 180 | 1 |
| 8 | K-180 5S | 180 | 5 |
| 9 | K-180 10S | 180 | 10 |
| 10 | K-180 15S | 180 | 15 |
| 11 | K-180 20S | 180 | 20 |
| 12 | K-200 1S | 200 | 1 |
| 13 | K-200 5S | 200 | 5 |
| 14 | K-200 10S | 200 | 10 |
| 15 | K-200 15S | 200 | 15 |
| 16 | K-200 20S | 200 | 20 |

**Fig. S1.** Representative base peak intensity (BPI) chromatograms for roasted laver extracts (QC) under positive ionization (**A**) and negative ionization (**B**). 69 metabolites, including 11 amino acids and their derivatives, 3 organic acids, 7 MAAs, 11 nucleic acid-related compounds, 24 phospholipids, 2 glycolipids, 5 other compounds, and 6 dehydrated MAAs were detected in roasted laver. These MS results are presented in Table 1.

**Fig. S2.** Base peak intensity (BPI) chromatograms of roasted laver samples obtained under positive ionization mode at different roasting temperatures and times. Roasting temperatures: 160, 180, and 200 °C; roasting times: 1, 5, 10, 15, and 20 s.

**Fig. S3.** ^1^H-(500 MHz) NMR spectrum of dehydrated porphyra-334 in D_2_O.

**Fig. S4.** MS/MS (positive ion mode) spectrum and fragmentation pathways of dehydrated aplysiapalythine B (**A**), dehydrated mycosporine-glycine-alanine (**B**), dehydrated asterina-330 (**C**), dehydrated shinorine (**D**), and dehydrated porphyra-334 (**E**) obtained from roasted laver.

**Fig. S5.** PCA score plot showing tight clustering of pooled QC samples (**A**) and comparison of RSD value distributions between experimental and QC samples (**B**).

**Fig. S6**. Validation of the ANOVA-simultaneous component analysis (ASCA) models for roasting temperature (**A**), time (**B**), and their interaction (**C**) using permutation tests (200 permutations). Leverage/SPE scatter plots of ASCA variable submodels for roasting temperature (**D**) and time (**E**) are also shown. In the leverage/SPE plots, metabolites in red have high leverage and low SPE, indicating a strong contribution to the major variation patterns captured by the corresponding submodels; metabolites in blue deviate from these major patterns.

**Fig. S7**. Venn diagram showing the numbers of metabolites significantly affected by roasting temperature, time, and their interaction, as determined by two-way ANOVA with Benjamini–Hochberg correction (*q* < 0.05).

**Table S2**. Influential metabolites associated with the main effects of roasting temperature and time, as identified by ASCA.

| Main effect | Metabolite | Leverage | SPE |
| --- | --- | --- | --- |
| Roasting temperature | Dehydrated porphyra-334 | 0.190 | 15.434 |
|  | Porphyra-334 | 0.109 | 20.649 |
|  | Dehydrated shinorine | 0.073 | 1.545 |
|  | Dehydrated palythine | 0.059 | 1.431 |
|  | Shinorine | 0.052 | 2.319 |
|  | Dehydrated asterina-330 | 0.052 | 0.863 |
|  | Dehydrated aplysiapalythine B | 0.040 | 0.657 |
|  | Pyroglutamic acid | 0.038 | 4.164 |
|  | Dehydrated mycosporine-glycine-alanine | 0.033 | 0.475 |
| Roasting time | Dehydrated porphyra-334 | 0.231 | 32.152 |
|  | Usujirene | 0.095 | 11.250 |
|  | Dehydrated shinorine | 0.074 | 7.004 |
|  | Shinorine | 0.060 | 48.453 |
|  | Dehydrated palythine | 0.058 | 28.392 |
|  | Dehydrated asterina-330 | 0.049 | 6.118 |
|  | Dehydrated aplysiapalythine B | 0.038 | 4.402 |
|  | Dehydrated mycosporine-glycine-alanine | 0.030 | 1.947 |

**Table S3**. Metabolites significantly affected by roasting temperature, time, and/or their interaction, according to two-way ANOVA in roasted laver.

| Metabolites | *q*-value | | |
| --- | --- | --- | --- |
|  | Roasting temperature | Roasting time | Interaction |
| Pyroglutamic acid | 9.38E-22 | 1.10E-20 | 1.70E-12 |
| Dehydrated mycosporine-glycine-alanine | 1.81E-20 | 1.10E-20 | 2.60E-09 |
| Dehydrated porphyra-334 | 3.17E-20 | 1.05E-21 | 1.06E-07 |
| Dehydrated shinorine | 3.17E-20 | 9.56E-21 | 1.82E-08 |
| Dehydrated asterina-330 | 5.22E-19 | 1.71E-19 | 1.06E-07 |
| Dehydrated aplysiapalythine B | 5.88E-19 | 1.84E-19 | 1.55E-07 |
| Guanosine | 3.15E-16 | 1.46E-14 | 1.36E-10 |
| 5'-IMP | 4.44E-15 | 1.09E-09 | 1.70E-12 |
| Shinorine | 6.68E-15 | 1.11E-16 | 1.12E-09 |
| Palythine | 1.21E-14 | 7.73E-16 | 1.12E-09 |
| Adenosine | 2.86E-14 | 2.27E-10 | 5.09E-06 |
| Dehydrated palythine | 7.48E-14 | 1.11E-14 | 1.17E-03 |
| Porphyra-334 | 1.40E-12 | 1.33E-14 | 5.38E-08 |
| Mycosporine-glycine-alanine | 2.16E-12 | 9.43E-15 | 1.10E-08 |
| Taurine | 9.06E-12 | 5.29E-01 | 7.10E-02 |
| Aplysiapalythine B | 3.43E-11 | 8.50E-14 | 2.07E-07 |
| Adenosine monophosphate | 4.26E-11 | 4.71E-11 | 3.58E-02 |
| Phycoerythrobilin | 4.26E-11 | 8.28E-15 | 1.11E-08 |
| Usujirene | 1.10E-10 | 1.89E-17 | 9.53E-04 |
| Cytidine | 1.26E-09 | 1.40E-08 | 1.33E-03 |
| Galactosylglycerol | 4.45E-09 | 4.60E-03 | 1.89E-01 |
| Uridine | 4.45E-09 | 8.90E-03 | 2.48E-02 |
| Inosine | 1.41E-06 | 5.71E-16 | 8.59E-08 |
| LPC 16:1 | 3.08E-06 | 8.05E-02 | 1.98E-01 |
| DGMG 16:0 | 4.22E-06 | 2.07E-01 | 2.53E-01 |
| 5'-GMP | 7.14E-06 | 7.27E-06 | 2.94E-04 |
| 5-Methylthioadenosine | 1.54E-05 | 1.67E-04 | 2.88E-01 |
| Asterina-330 | 2.05E-05 | 5.45E-09 | 3.97E-04 |
| LPC 20:2 | 1.19E-04 | 5.29E-01 | 1.12E-01 |
| LPC 20:1 | 2.55E-04 | 4.56E-01 | 3.21E-01 |
| LPC 18:2 | 2.70E-04 | 6.53E-01 | 2.41E-01 |
| LPC 20:3 | 2.70E-04 | 6.63E-01 | 2.27E-01 |
| Glycerophosphocholine | 2.82E-04 | 8.07E-01 | 3.29E-01 |
| 2,6-Piperidinedicarboxylic acid | 3.39E-04 | 5.93E-01 | 2.70E-01 |
| Alanine | 3.63E-04 | 7.73E-01 | 1.89E-01 |
| LPC 18:3 | 4.95E-04 | 6.47E-01 | 2.53E-01 |
| LPC 16:0 | 5.15E-04 | 6.21E-01 | 1.98E-01 |
| LPC 20:4 | 5.92E-04 | 5.29E-01 | 2.03E-01 |
| LPC 14:0 | 6.39E-04 | 6.62E-01 | 1.72E-01 |
| LPC 18:1 | 9.23E-04 | 6.23E-01 | 2.67E-01 |
| LPG 20:4 | 1.09E-03 | 3.36E-01 | 2.03E-01 |
| SQMG 16:0 | 1.53E-03 | 6.21E-01 | 2.23E-01 |
| LPC 18:4 | 1.53E-03 | 6.62E-01 | 3.03E-01 |
| LPE 20:5 | 1.53E-03 | 2.03E-02 | 5.98E-02 |
| LPE 18:2 | 1.53E-03 | 2.42E-01 | 1.53E-01 |
| Acetylcholine | 3.00E-03 | 6.16E-01 | 2.53E-01 |
| LPC 20:5 | 4.40E-03 | 5.70E-01 | 3.03E-01 |
| Choline | 6.90E-03 | 6.16E-01 | 1.98E-01 |
| LPE 20:4 | 6.90E-03 | 6.02E-01 | 8.05E-02 |
| LPE 20:3 | 6.90E-03 | 6.31E-02 | 3.70E-02 |
| Glutamic acid | 1.08E-02 | 9.69E-07 | 3.46E-04 |
| Tyrosine | 1.73E-02 | 4.62E-01 | 2.53E-01 |
| LPE 16:0 | 2.08E-02 | 3.36E-01 | 5.71E-02 |
| Tryptophan | 2.56E-02 | 4.41E-01 | 2.10E-01 |
| Aspartic acid | 2.63E-02 | 1.29E-02 | 1.90E-01 |
| LPE 16:1 | 2.66E-02 | 4.60E-01 | 1.89E-01 |
| LPG 20:5 | 2.66E-02 | 4.56E-01 | 2.53E-01 |
| Citric acid | 4.52E-02 | 1.46E-02 | 6.04E-02 |
| 5'-UMP | 1.37E-01 | 1.88E-04 | 6.27E-02 |
| 5'-CMP | 1.52E-01 | 1.73E-04 | 6.04E-02 |
| LPI 20:5 | 3.48E-01 | 1.83E-07 | 1.44E-05 |
| Citrulline | 4.97E-01 | 4.60E-03 | 3.18E-02 |
| LPA 20:5 | 7.76E-01 | 6.88E-04 | 7.10E-04 |

5′-CMP, cytidine 5′-monophosphate; 5′-UMP, 5′-uridine monophosphate; 5′-AMP, 5′-adenosine monophosphate, 5′-GMP, 5′-guanosine monophosphate; 5′-IMP, inosine 5′-monophosphate; LPC, lysophosphatidylcholine; LPE, lysophosphatidylethanolamine; LPA, lysophosphatidic acid; LPG, lysophosphatidylglycerol; LPI, lysophosphatidylinositol; DGMG, digalactosylmonoacylglycerol; SQMG, sulfoquinovosylmonoacylglycerol.

**Fig. S8.** Changes in the contents of seven MAAs in roasted laver. Roasting temperatures: 160, 180, and 200 °C; roasting times: 1, 5, 10, 15, and 20 s. Values are presented as mean ± standard error (n = 3). Different uppercase letters (A–D) within the same roasting temperature indicate significant differences among roasting times (*p* < 0.05); different lowercase letters (a–c) within the same roasting time indicate significant differences among roasting temperatures (*p* < 0.05), according to Tukey’s multiple comparison test. Asterisks (*) indicate significant differences between each roasted treatment and the unroasted control according to Dunnett’s test (*p* < 0.05). MAA, mycosporine-like amino acid.

**Table S4.** Partial eta squared (η_p_^2^) values for roasting temperature, time, and their interaction for seven MAAs and six dehydrated MAAs in roasted laver.

| Class | Compounds | Temp | Time | Temp.×Time |
| --- | --- | --- | --- | --- |
| Mycosporine-like amino acids | Shinorine | 0.809^***^ | 0.895^***^ | 0.747^***^ |
|  | Palythine | 0.829^***^ | 0.890^***^ | 0.791^***^ |
|  | Asteria-330 | 0.421^***^ | 0.721^***^ | 0.549^***^ |
|  | Porphyra-334 | 0.748^***^ | 0.858^***^ | 0.691^***^ |
|  | Mycosporine-glycine-alanine | 0.699^***^ | 0.850^***^ | 0.706^***^ |
|  | Aplysiapalythine B | 0.673^***^ | 0.835^***^ | 0.664^***^ |
|  | Usujirene | 0.661^***^ | 0.922^***^ | 0.468^***^ |
|  | Dehydrated palythine | 0.863^***^ | 0.867^***^ | 0.636^***^ |
| Dehydrated Mycosporine-like amino acids | Dehydrated asteria-330 | 0.925^***^ | 0.918^***^ | 0.808^***^ |
|  | Dehydrated aplysiapalythine B | 0.925^***^ | 0.920^***^ | 0.802^***^ |
|  | Dehydrated shinorine | 0.940^***^ | 0.939^***^ | 0.839^***^ |
|  | Dehydrated porphyra-334 | 0.954^***^ | 0.963^***^ | 0.834^***^ |
|  | Dehydrated mycosporine-glycine-alanine | 0.944^***^ | 0.933^***^ | 0.864^***^ |

^∗∗∗^ indicates *p* < 0.001.

**Table S5.** Pearson’s correlation coefficients between antioxidant activity and 69 metabolites detected in roasted laver.

| Compounds | ABTS^+^ | | | FRAP | | |
| --- | --- | --- | --- | --- | --- | --- |
|  | *r* | *q*-value | 95% CI | *r* | *q*-value | 95% CI |
| Dehydrated porphyra-334 | 0.98 | 1.32E-36 | 0.9646 to 0.9889 | 0.98 | 1.04E-31 | 0.9588 to 0.9870 |
| Dehydrated shinorine | 0.95 | 6.68E-32 | 0.9161 to 0.9732 | 0.97 | 1.29E-33 | 0.9423 to 0.9817 |
| Dehydrated aplysiapalythine B | 0.95 | 3.22E-31 | 0.9092 to 0.9709 | 0.96 | 4.57E-32 | 0.9310 to 0.9780 |
| Dehydrated asterina-330 | 0.94 | 3.93E-31 | 0.9029 to 0.9688 | 0.96 | 4.57E-32 | 0.9273 to 0.9768 |
| Dehydrated mycosporine-glycine-alanine | 0.93 | 3.61E-29 | 0.8809 to 0.9614 | 0.96 | 1.08E-33 | 0.9282 to 0.9771 |
| Dehydrated palythine | 0.93 | 3.96E-24 | 0.8722 to 0.9585 | 0.93 | 2.92E-24 | 0.8830 to 0.9621 |
| 5'-AMP | 0.90 | 2.00E-20 | 0.8271 to 0.9429 | 0.91 | 2.62E-20 | 0.8403 to 0.9475 |
| Pyroglutamic acid | 0.84 | 4.26E-17 | 0.7318 to 0.9082 | 0.91 | 1.23E-23 | 0.8515 to 0.9514 |
| Adenosine | 0.82 | 2.39E-12 | 0.6918 to 0.8929 | 0.89 | 2.00E-16 | 0.8085 to 0.9363 |
| Phycoerythrobilin | 0.81 | 6.81E-16 | 0.6839 to 0.8898 | 0.72 | 5.93E-12 | 0.5545 to 0.8365 |
| Cytidine | 0.81 | 1.34E-11 | 0.6791 to 0.8879 | 0.87 | 1.93E-14 | 0.7756 to 0.9245 |
| Guanosine | 0.78 | 1.35E-10 | 0.6396 to 0.8721 | 0.87 | 2.60E-14 | 0.7727 to 0.9234 |
| Galactosylglycerol | 0.76 | 2.67E-09 | 0.6068 to 0.8587 | 0.79 | 1.38E-10 | 0.6503 to 0.8765 |
| 5'-Methylthioadenosine | 0.74 | 1.38E-09 | 0.5729 to 0.8444 | 0.72 | 4.09E-09 | 0.5546 to 0.8365 |
| 5'-GMP | 0.71 | 6.39E-08 | 0.5292 to 0.8254 | 0.68 | 1.37E-07 | 0.4900 to 0.8077 |
| Uridine | 0.69 | 5.01E-07 | 0.5113 to 0.8174 | 0.77 | 3.58E-09 | 0.6251 to 0.8662 |
| LPC 16:1 | 0.68 | 9.82E-07 | 0.4845 to 0.8052 | 0.70 | 1.49E-07 | 0.5210 to 0.8217 |
| Taurine | 0.63 | 9.65E-06 | 0.4227 to 0.7761 | 0.68 | 6.49E-07 | 0.4896 to 0.8076 |
| LPC 20:1 | 0.60 | 1.73E-05 | 0.3773 to 0.7536 | 0.60 | 1.22E-05 | 0.3788 to 0.7544 |
| DGMG 16:0 | 0.60 | 4.64E-05 | 0.3830 to 0.7565 | 0.64 | 6.08E-06 | 0.4374 to 0.7832 |
| LPC 20:2 | 0.53 | 3.21E-04 | 0.2873 to 0.7065 | 0.54 | 2.09E-04 | 0.2958 to 0.7111 |
| 5'-CMP | 0.53 | 1.05E-04 | 0.2898 to 0.7078 | 0.45 | 9.92E-04 | 0.1950 to 0.6538 |
| 5'-UMP | 0.50 | 3.15E-04 | 0.2545 to 0.6883 | 0.43 | 1.82E-03 | 0.1706 to 0.6392 |
| LPC 20:3 | 0.45 | 3.65E-03 | 0.1958 to 0.6543 | 0.48 | 1.52E-03 | 0.2262 to 0.6722 |
| LPC 18:2 | 0.44 | 4.24E-03 | 0.1826 to 0.6465 | 0.46 | 2.26E-03 | 0.2033 to 0.6588 |
| LPC 18:1 | 0.44 | 4.88E-03 | 0.1736 to 0.6410 | 0.45 | 2.60E-03 | 0.1953 to 0.6541 |
| LPC 16:0 | 0.44 | 5.59E-03 | 0.1750 to 0.6418 | 0.46 | 2.60E-03 | 0.2041 to 0.6592 |
| 2,6-Piperidinedicarboxylic acid | 0.42 | 7.05E-03 | 0.1526 to 0.6281 | 0.44 | 3.73E-03 | 0.1810 to 0.6455 |
| LPC 18:3 | 0.41 | 7.49E-03 | 0.1440 to 0.6228 | 0.42 | 4.83E-03 | 0.1568 to 0.6307 |
| LPC 20:4 | 0.41 | 8.23E-03 | 0.1474 to 0.6249 | 0.44 | 3.96E-03 | 0.1789 to 0.6442 |
| LPI 20:5 | 0.41 | 1.05E-03 | 0.1450 to 0.6234 | 0.32 | 8.84E-03 | 0.03904 to 0.5536 |
| LPC 14:0 | 0.37 | 2.29E-02 | 0.09367 to 0.5905 | 0.39 | 1.16E-02 | 0.1246 to 0.6105 |
| Glycerophosphocholine | 0.36 | 2.59E-02 | 0.08442 to 0.5844 | 0.40 | 1.08E-02 | 0.1295 to 0.6137 |
| LPC 20:5 | 0.35 | 3.02E-02 | 0.07064 to 0.5752 | 0.37 | 1.89E-02 | 0.09485 to 0.5913 |
| LPC 18:4 | 0.31 | 4.91E-02 | 0.03258 to 0.5491 | 0.33 | 3.79E-02 | 0.04710 to 0.5592 |
| Acetylcholine | 0.29 | 7.66E-02 | 0.0008941 to 0.5266 | 0.30 | 5.91E-02 | 0.02106 to 0.5410 |
| LPA 20:5 | 0.29 | 1.61E-02 | 0.009531 to 0.5328 | 0.18 | 1.01E-01 | −0.1110 to 0.4407 |
| LPE 20:4 | 0.25 | 1.17E-01 | −0.03269 to 0.5019 | 0.25 | 1.08E-01 | −0.03688 to 0.4987 |
| Alanine | 0.24 | 1.59E-01 | −0.05003 to 0.4888 | 0.29 | 7.64E-02 | 0.008641 to 0.5321 |
| Choline | 0.22 | 1.59E-01 | −0.06549 to 0.4769 | 0.22 | 1.61E-01 | −0.07291 to 0.4711 |
| SQMG 16:0 | 0.21 | 2.70E-01 | −0.08087 to 0.4648 | 0.27 | 1.42E-01 | −0.01760 to 0.5131 |
| Tryptophan | 0.19 | 2.58E-01 | −0.1003 to 0.4494 | 0.19 | 2.53E-01 | −0.1016 to 0.4483 |
| Tyrosine | 0.18 | 2.70E-01 | −0.1079 to 0.4432 | 0.19 | 2.53E-01 | −0.1023 to 0.4477 |
| LPE 16:1 | 0.11 | 4.27E-01 | −0.1819 to 0.3804 | 0.05 | 6.60E-01 | −0.2348 to 0.3321 |
| Succinic acid | 0.01 | 8.93E-01 | −0.2745 to 0.2938 | −0.04 | 8.28E-01 | −0.3177 to 0.2500 |
| Malic acid | −0.04 | 7.90E-01 | −0.3176 to 0.2501 | −0.06 | 6.41E-01 | −0.3400 to 0.2264 |
| Glycerophosphoglycerol | −0.06 | 7.25E-01 | −0.3381 to 0.2285 | −0.09 | 5.79E-01 | −0.3610 to 0.2035 |
| Isethionic acid | −0.14 | 3.59E-01 | −0.4092 to 0.1487 | −0.16 | 2.59E-01 | −0.4285 to 0.1257 |
| LPE 16:0 | −0.22 | 2.03E-01 | −0.4723 to 0.07131 | −0.30 | 6.36E-02 | −0.5378 to −0.01651 |
| LPE 18:2 | −0.23 | 1.52E-01 | −0.4806 to 0.06069 | −0.27 | 7.64E-02 | −0.5143 to 0.01597 |
| LPG 20:5 | −0.27 | 6.28E-02 | −0.5155 to 0.01426 | −0.26 | 6.24E-02 | −0.5106 to 0.02092 |
| Aspartic acid | −0.28 | 4.82E-02 | −0.5257 to 0.0003285 | −0.22 | 1.08E-01 | −0.4764 to 0.06606 |
| LPG 16:1 | −0.32 | 2.51E-02 | −0.5569 to −0.04379 | −0.33 | 2.25E-02 | −0.5583 to −0.04578 |
| Citrulline | −0.34 | 2.32E-02 | −0.5680 to −0.06003 | −0.38 | 1.07E-02 | −0.5958 to −0.1018 |
| LPG 16:0 | −0.39 | 7.49E-03 | −0.6098 to −0.1235 | −0.39 | 6.80E-03 | −0.6066 to −0.1185 |
| LPE 20:3 | −0.40 | 1.06E-02 | −0.6132 to −0.1289 | −0.46 | 2.00E-03 | −0.6611 to −0.2072 |
| LPE 20:5 | −0.44 | 4.60E-03 | −0.6422 to −0.1756 | −0.50 | 7.95E-04 | −0.6846 to −0.2480 |
| LPG 20:4 | −0.47 | 1.05E-03 | −0.6644 to −0.2128 | −0.45 | 1.39E-03 | −0.6529 to −0.1934 |
| Citric acid | −0.49 | 9.06E-04 | −0.6817 to −0.2429 | −0.52 | 2.77E-04 | −0.7011 to −0.2776 |
| Inosine | −0.61 | 3.66E-07 | −0.7594 to −0.3887 | −0.53 | 1.22E-05 | −0.7110 to −0.2956 |
| Glutamic acid | −0.62 | 1.73E-05 | −0.7655 to −0.4010 | −0.66 | 1.32E-06 | −0.7981 to −0.4690 |
| 5'-IMP | −0.64 | 6.97E-06 | −0.7827 to −0.4364 | −0.73 | 6.45E-08 | −0.8384 to −0.5590 |
| Asterina-330 | −0.81 | 2.64E-11 | −0.8870 to −0.6768 | −0.81 | 1.25E-12 | −0.8911 to −0.6872 |
| Mycosporine-glycine-alanine | −0.89 | 8.75E-14 | −0.9374 to −0.8115 | −0.90 | 1.88E-17 | −0.9457 to −0.8351 |
| Aplysiapalythine B | −0.89 | 3.63E-15 | −0.9397 to −0.8181 | −0.90 | 1.51E-18 | −0.9440 to  −0.8302 |
| Usujirene | −0.89 | 2.04E-22 | −0.9367 to −0.8097 | −0.84 | 4.11E-20 | −0.9100 to −0.7367 |
| Porphyra-334 | −0.91 | 2.88E-15 | −0.9474 to −0.8401 | −0.92 | 5.99E-19 | −0.9531 to −0.8565 |
| Palythine | −0.91 | 2.64E-15 | −0.9512 to −0.8509 | −0.93 | 2.97E-19 | −0.9601 to −0.8769 |
| Shinorine | −0.93 | 8.81E-16 | −0.9583 to −0.8717 | −0.94 | 1.52E-19 | −0.9644 to −0.8899 |

n = 48; CI, 95% confidence intervals; 5′-CMP, cytidine 5′-monophosphate; 5′-UMP, 5′-uridine monophosphate; 5′-AMP, 5′-adenosine monophosphate, 5′-GMP, 5′-guanosine monophosphate; 5′-IMP, inosine 5′-monophosphate; LPC, lysophosphatidylcholine; LPE, lysophosphatidylethanolamine; LPA, lysophosphatidic acid; LPG, lysophosphatidylglycerol; LPI, lysophosphatidylinositol; DGMG, digalactosylmonoacylglycerol; SQMG, sulfoquinovosylmonoacylglycerol.
